# A reductive coupling strategy towards ripostatin A

**DOI:** 10.3762/bjoc.9.175

**Published:** 2013-07-31

**Authors:** Kristin D Schleicher, Timothy F Jamison

**Affiliations:** 1 Department of Chemistry, Massachusetts Institute of Technology, 77 Massachusetts Ave., Cambridge, MA 02139, United States

**Keywords:** catalysis, natural product, nickel, reductive coupling, ripostatin A, synthesis

## Abstract

Synthetic studies on the antibiotic natural product ripostatin A have been carried out with the aim to construct the C9−C10 bond by a nickel(0)-catalyzed coupling reaction of an enyne and an epoxide, followed by rearrangement of the resulting dienylcyclopropane intermediate to afford the skipped 1,4,7-triene. A cyclopropyl enyne fragment corresponding to C1−C9 has been synthesized in high yield and demonstrated to be a competent substrate for the nickel(0)-catalyzed coupling with a model epoxide. Several synthetic approaches toward the C10−C26 epoxide have been pursued. The C13 stereocenter can be set by allylation and reductive decyanation of a cyanohydrin acetonide. A mild, fluoride-promoted decarboxylation enables construction of the C15−C16 bond by an aldol reaction. The product of this transformation is of the correct oxidation state and potentially three steps removed from the targeted epoxide fragment.

## Introduction

**Figure 1:**
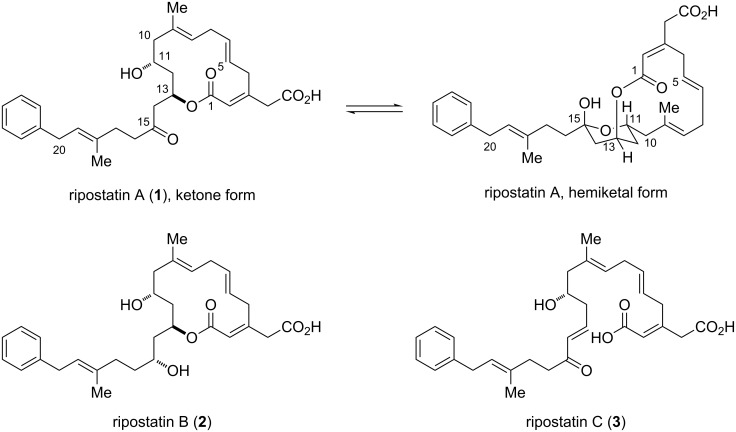
Structures of the ripostatins.

The ripostatins (A, B, and C) are a family of antibiotic natural products, isolated in 1995 by Höfle and colleagues from cultures of the myxobacterium *Sorangium cellulosum* (Figure 1) [1–2]. Ripostatins A and B are active against Gram-positive bacteria due to their inhibition of bacterial ribonucleic acid polymerase. These compounds inhibit chain initiation of RNA synthesis in *Staphylococcus aureus,* a particularly infectious bacterial strain with reported drug resistance to the antibiotics vancomycin and methicillin [3]. Ripostatin A exists as an equilibrium mixture of ketone and hemiketal forms, the ratio of which is reported to be 55:45 in methanolic solution. In the hemiketal form, the bicyclic framework of ripostatin A features an unusual *in*/*out* connectivity. The 14-membered macrocyclic core of ripostatin A contains three double bonds, arranged in a rare 1,4,7-skipped triene. The alkene geometry was determined to be (2*E*,5*E*,8*E*) by measurement of NOE enhancements [4]. Ripostatin B and its C15 epimer can be obtained from ripostatin A by reduction with sodium borohydride, while ripostatin C can be formed from ripostatin A by a mild base-mediated elimination. Consequently, ripostatin A was selected as the primary target for synthesis.

**Figure 2:**
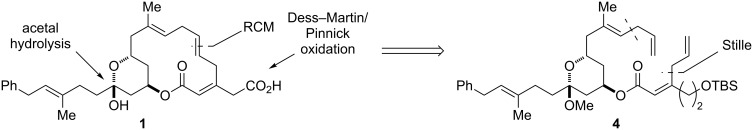
Retrosynthesis of ripostatin A.

At the outset of our efforts, the only published synthetic study on the ripostatins was Kirschning’s synthesis of C1−C5 and C6−C24 fragments of ripostatin B [5]. However, these fragments could not be connected by esterification due to steric hindrance from bulky protecting groups, as well as susceptibility of the skipped dienes therein to double bond isomerization and migration under basic conditions. However, the ripostatins intrigued others, and three total syntheses of ripostatin B were published in succession in 2012 [6–8]. Tang and Prusov extended their synthetic method to syntheses of 15-deoxyripostatin A, and later ripostatin A itself [9]. All of these approaches to the ripostatins share several key features: use of ring-closing metathesis to form the 14-membered macrocycle, preceded by one or more Stille couplings to generate the double 1,4-diene (Figure 2).

Notwithstanding the successful syntheses of ripostatins, preparation of configurationally defined skipped polyenes (1,4-dienes and higher homologues) remains a significant challenge in organic chemistry. The doubly allylic protons found in these structures may be sensitive to strong base as well as hydrogen abstraction [10–11]. While classical methods for the preparation of 1,4-dienes include partial reduction of alkynes and carbonyl olefination, a variety of transition-metal-mediated processes have been developed for the synthesis of skipped dienes of varied substitution patterns [12–15]. Most recently, Sigman and colleagues have reported a palladium-catalyzed 1,4-difunctionalization of 1,4-butadiene with vinylboronic acid and vinyl triflate that can be used to rapidly access the skipped triene of ripostatin A [16].

**Scheme 1:**
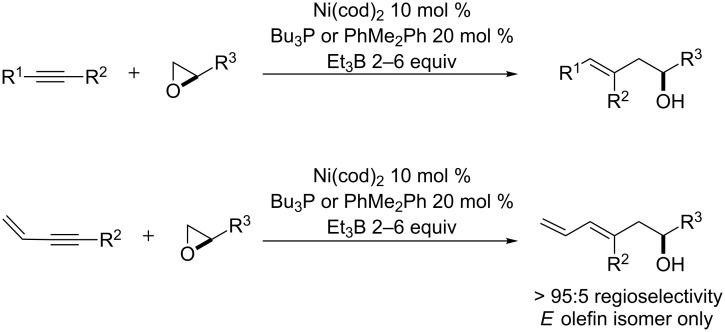
Nickel-catalyzed reductive coupling of alkynes and epoxides.

We recognized that the C11 stereocenter and the C8–C9 trisubstituted olefin of ripostatin A mapped onto a nickel-catalyzed coupling reaction of alkynes and epoxides developed in our laboratory (Scheme 1) [17]. In the intermolecular reaction, stereospecific *cis* addition across the alkyne is observed, and the stereochemistry at the epoxide is preserved in the transformation. Aliphatic epoxides are opened selectively (>95:5) at the terminal position. Although very high regioselectivity with respect to the alkyne is observed when R^1^ = Ph and R^2^ = Me, attempts to differentiate between aliphatic alkyne substituents lead to mixture of regioisomers. When a 1,3-enyne is coupled with simple epoxides, however, >95:5 regioselectivity is observed for C–C bond formation distal to the pendant alkene [18].

**Figure 3:**
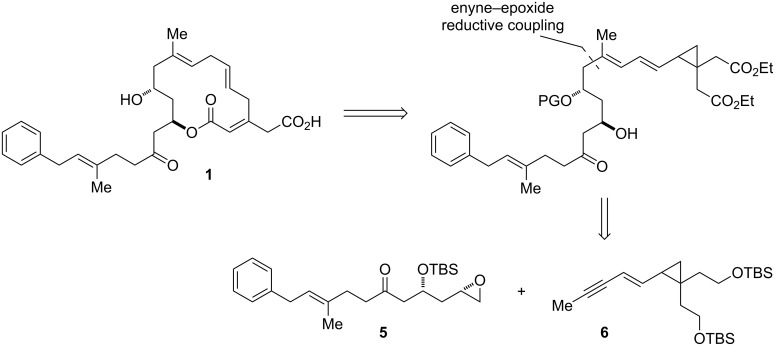
Proposed retrosynthesis of ripostatin A featuring enyne–epoxide reductive coupling and rearrangement.

In conjunction with the nickel-catalyzed fragment coupling, we wished to investigate whether it would be possible to delay introduction of the potentially sensitive 1,4,7-triene by masking it as a cyclopropyldiene, then unveiling the skipped triene portion via a 1,5-hydrogen rearrangement (Figure 3). This strategy would allow us to take advantage of the high regioselectivity in enyne–epoxide reductive coupling reactions. Furthermore, the proposed rearrangement would serve to differentiate the ester groups, as hydrogen would migrate from adjacent to the ester *cis* to the dienyl chain only.

**Scheme 2:**
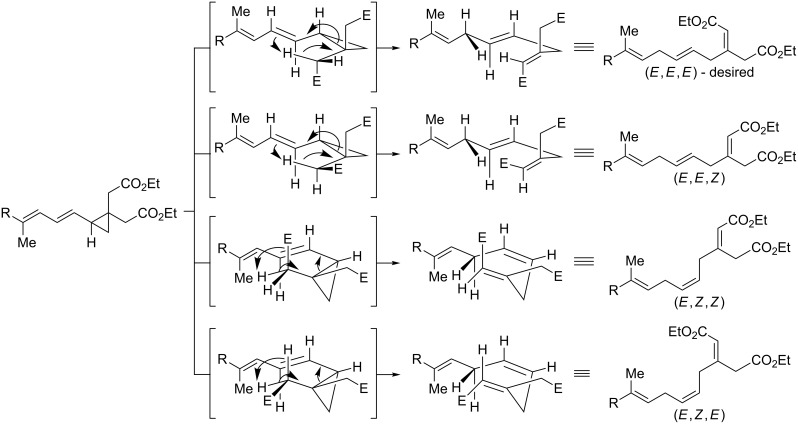
Potential transition states and stereochemical outcomes for a concerted 1,5-hydrogen rearrangement.

While offering a unique approach to the skipped triene portion of ripostatin, this route was not without significant uncertainty. First, substrates containing a vinyl cyclopropane unit had not been previously tested under nickel-catalyzed reductive coupling conditions. Nickel(0) is known to catalyze the rearrangement of vinylcyclopropanes to cyclopentenes; however, activating substituents are commonly required [19–21]. Furthermore, application of a proposed 1,5-hydrogen rearrangement to ripostatin A would require that the reaction proceed to give the triene with *E,E,E* configuration selectively out of four possible configurational outcomes (Scheme 2).

**Scheme 3:**
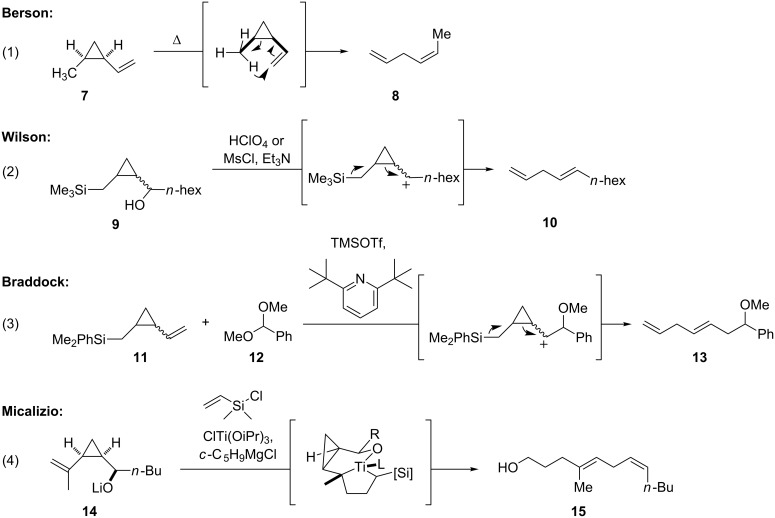
Rearrangements of vinylcyclopropanes to acylic 1,4-dienes.

Rearrangement of vinylcyclopropanes has been used to prepare 1,4-skipped dienes of varying geometry (Scheme 3). It has long been known that *cis*-disubstituted vinylcyclopropanes can undergo 1,5-hydrogen migration under thermal conditions to deliver acyclic 1,4-dienes [22]. In this reaction manifold, the new “acceptor-derived” double bond is formed via an endo transition state, leading to a *cis* olefinic configuration in the product (Scheme 3, reaction 1). Berson has quantified the energetic preference in this transformation, while Turos has shown that the presence of a silicon substituent on the “donor carbon” facilitates the hydrogen migration [23–26].

In contrast, Wilson has reported formation of the *trans* double bond in the opening of a cyclopropane with an adjacent hydroxy or mesylate leaving group, regardless of the initial configuration of the cyclopropane (Scheme 3, reaction 2) [27]. Braddock has demonstrated that the internal 3,4-*E* olefin is obtained exclusively in Prins reactions terminated by cyclopropylmethylsilane (Scheme 3, reaction 3), which may be explained by the participation of a carbocation that is stabilized by the adjacent cyclopropane ring in the bisected conformation where (CHOR)R′ is oriented *anti* to the cyclopropane [28–30]. Finally, Micalizio has described a titanium-mediated, alkoxide-directed fragment coupling reaction between vinylcylopropanes and vinyldimethylchlorosilane (Scheme 3, reaction 4) in which the stereochemical outcome of the rearrangement is orchestrated by the adjacent alkoxide, which is believed to direct formation of a tricyclic titanacyclopentane that subsequently fragments in a stereospecific manner [31].

We were intrigued by the apparent difference in selectivity observed in cyclopropane rearrangements proceeding via a neutral pathway versus those proceeding by more polar or directed mechanisms. Although the *E* geometry of the central olefin in the ripostatin A triene is more consistent with a polar mode of reactivity than a neutral 1,5-hydrogen migration, we still wished to investigate the outcome of rearrangement under thermal conditions. To the best of our knowledge, such a rearrangement has not been explored for structures containing an additional alkenyl substituent in conjugation, or with electron-withdrawing groups adjacent to the site of hydrogen migration. In particular, the latter’s ability to facilitate the buildup of negative charge at an adjacent carbon might play an important role in the stereoelectronic course of the reaction.

## Results and Discussion

### Synthesis of cyclopropylenyne and reductive coupling with model epoxide

**Scheme 4:**
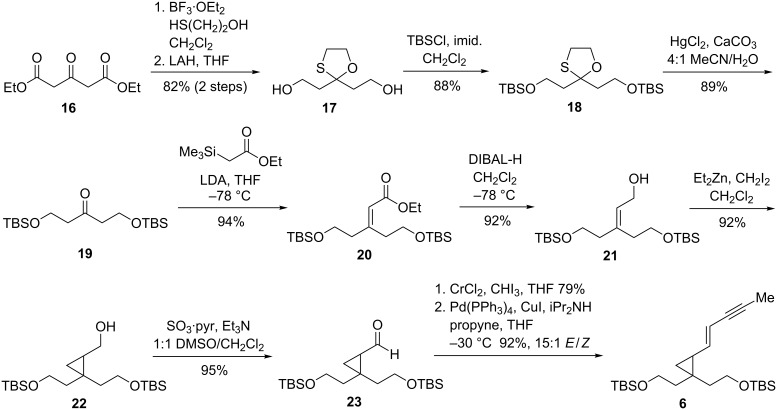
Synthesis of cyclopropyl enyne.

Diethyl 1,3-acetonedicarboxylate (Scheme 4, **16**) was rapidly identified as an inexpensive five-carbon fragment possessing the appropriate oxygenation pattern for preparation of the C1–C9 enyne fragment. However, due to keto–enol tautomerization, carbonyl olefination methods are of limited utility for this substrate. Instead, the ketone was protected as the mixed *S*,*O*-ketal and reduced to the diol **17**. Protection of the hydroxy groups and removal of the ketal afforded ketone **19**. A number of alternative promoters were investigated to avoid the use of mercury(II) salts in the ketal deprotection (including MeI, H_2_O_2_, AgClO_4_/I_2_); however, these generally led to concomitant removal of the TBS groups.

Ketone **19** was converted to the α,β-unsaturated ester **20** using the Peterson olefination [32]. Treatment of **20** with the sulfur ylide derived from trimethylsulfoxonium iodide [33–34] led to recovery of starting material at room temperature, but decomposition at elevated temperatures. Instead, the enone was smoothly reduced to the allylic alcohol, and a Furukawa-modified Simmons–Smith reaction [35] afforded the cyclopropyl alcohol **22** in high yield.

Oxidation to the cyclopropyl aldehyde **23** offered a branching point from which either the *E*- or *Z*-substituted enyne could be synthesized, should we wish to study the rearrangement of both diene geometries. To access our initial target, the *E*-enyne **6**, a Takai olefination [36] was used to generate the *E*-vinyl iodide. The vinyl iodide was sufficiently stable for purification by silica gel chromatography but, following purification, was immediately carried forward to a Sonogashira reaction with propyne [37]. The enyne could be obtained in 15:1 *E*/*Z* selectivity, in 10 steps and 35% overall yield. It was found that the use of freshly distilled THF in the Takai olefination and careful temperature control in the subsequent cross coupling were critical to the preservation of high *E*/*Z* selectivity over the course of these transformations. Once isolated, however, enyne **6** proved to be quite stable and could be stored for extended periods at 0–5 °C without appreciable isomerization or decomposition.

**Scheme 5:**
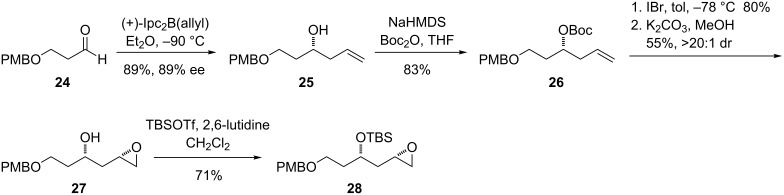
Synthesis of model epoxide for investigation of the nickel-catalyzed coupling reaction.

With a suitable cyclopropylenyne in hand, a model epoxide substrate containing the 1,3-oxygenation pattern found in ripostatin A (Scheme 5) was prepared by using a route analogous to one reported by Smith [38]. Allylation of **24** with (+)-*B*-allyldiisopinocampheylborane generated the alcohol **25** in high yield and enantioselectivity. Directed epoxidation using VO(acac)_2_ and *tert*-butyl hydroperoxide was initially performed in order to furnish **27** directly; however, this proceeded in only modest yield and diastereoselectivity (53%, 2.8:1 *syn*/*anti*). Although the ratio of *syn*/*anti* epoxide diastereomers could be enhanced by subjecting the mixture to hydrolytic kinetic resolution [39], greater throughput could be obtained by converting **25** to the *tert*-butyl carbonate, performing an iodocyclization, then cleaving the iodocarbonate and closing the epoxide under basic conditions. Silyl protection of the secondary alcohol afforded the desired model compound **28**.

**Scheme 6:**
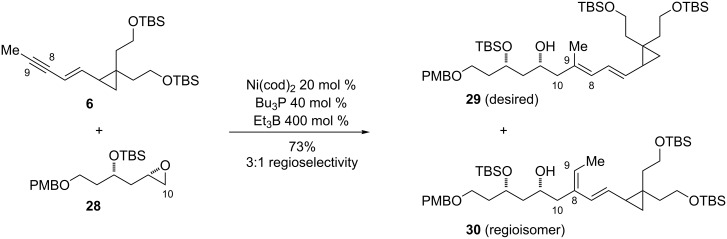
Nickel-catalyzed enyne–epoxide reductive coupling reaction.

Enyne **6** and epoxide **28** were subjected to standard nickel-catalyzed reductive coupling conditions, and reductive coupling proceeded in good yield, leaving the cyclopropane ring intact (Scheme 6). However, the desired diene **29** was isolated along with the regioisomeric product **30** in approximately a 3:1 ratio. The desired product could be partially separated from the regioisomer by careful silica gel chromatography.

**Scheme 7:**
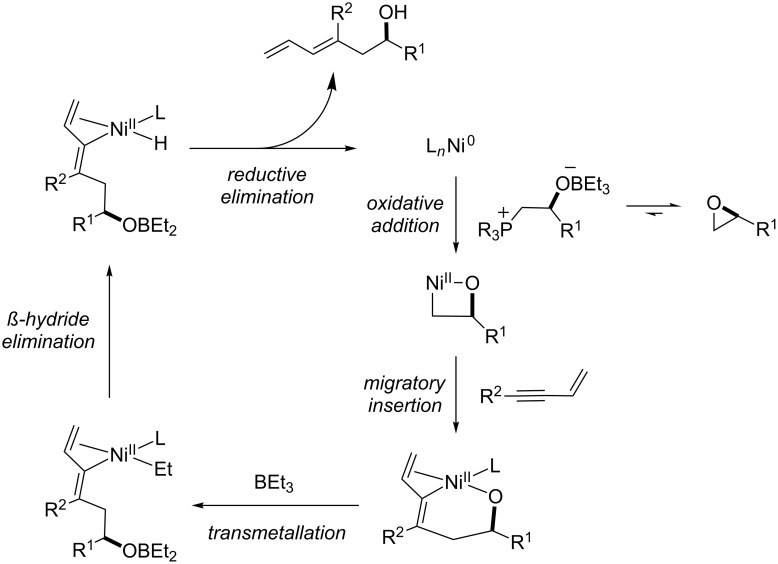
Proposed mechanism for the nickel-catalyzed coupling reaction of alkynes or enynes with epoxides.

It may be instructive at this stage to consider the proposed mechanism of the nickel-catalyzed alkyne or enyne–epoxide reductive coupling reaction (Scheme 7). In contrast to the mechanistic framework proposed [40–41] for reductive coupling reactions of alkynes and aldehydes developed in our laboratory [42–43], it is believed that epoxide oxidative addition precedes alkyne addition, as opposed to concerted oxidative coupling. At least when dimethylphenylphosphine is used as ligand, this may proceed via the intermediacy of a betaine species. In the reductive coupling reaction of enynes and epoxides, the olefin coordinates to nickel and directs alkyne insertion.

**Scheme 8:**
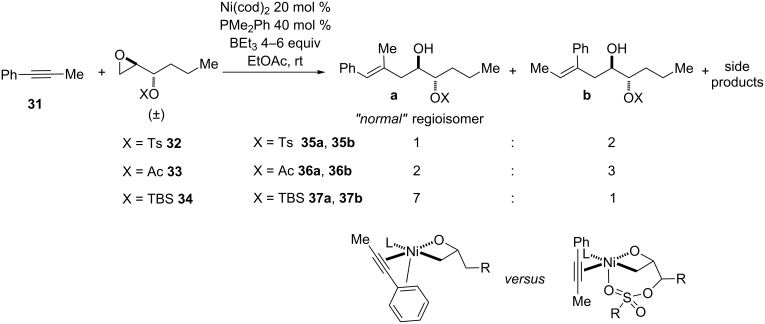
Regioselectivity changes in reductive couplings of alkynes and 3-oxygenated epoxides.

Because of this directing effect, formation of the regioisomeric diene product is atypical for reductive coupling reactions of enynes and epoxides. However, in reactions of 1-phenyl-1-propyne and epoxides with oxygenation in the 3-position (Scheme 8), it was found that while epoxides containing adjacent silyl ethers afforded mainly the expected regioisomer (7:1 **37a**/**37b**), epoxides with sulfonate esters (e.g., tosyl, **32**) and esters (e.g., acetyl, **33**) afforded a regioisomeric mixture of opposite (albeit poor) selectivity relative to that normally observed for unfunctionalized epoxides [44]. This is proposed to be an effect of the coordinating ability of Lewis basic oxygen atoms in tosylates and esters, which may disrupt the binding and directing effect of phenyl or alkenyl groups.

**Scheme 9:**
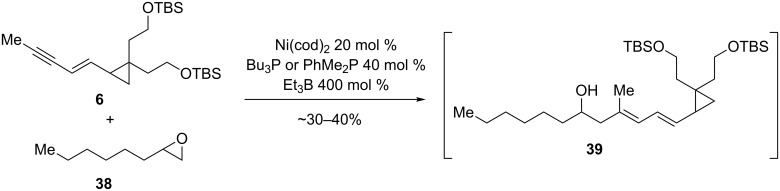
Enyne reductive coupling with 1,2-epoxyoctane.

In the case of the ripostatin A model system, the most likely candidate for chelation is the oxygen protected as the PMB ether. Although an eight-membered chelate might seem too large to play an important role in directing regioselectivity, the 3:1 regioselectivity observed in the “normal” direction is consistent with chelation playing a diminished role relative to the seven-membered chelates invoked for coupling of 3-oxygenated epoxides. We attempted to discern whether this interaction was the reason for the observed regioselectivity by performing the nickel-catalyzed coupling reaction with 1,2-epoxyoctane, which lacks potentially chelating functional groups (Scheme 9). Although the coupling product **39** appears to be formed in the reaction with either Bu_3_P or PhMe_2_P as ligand, the non-polar nature of this molecule complicates chromatographic purification, and mixtures of what appears to be **39** along with one or more other products were obtained. Based on these results, regioisomer formation cannot be excluded.

Several aspects of the enyne synthesis and the nickel-catalyzed coupling reaction require further investigation. As **6** does not itself appear to undergo thermal rearrangement, it seems advantageous to convert this compound to the corresponding diester. Preliminary investigation indicates that oxidation of the diol derived from **6** is complicated by the 1,5-relationship of the alcohols. Despite the greater complexity inherent to this alternative, differentiation of the alcohols allowing for sequential oxidation may be necessary.

### Dithiane approach to epoxide

**Figure 4:**
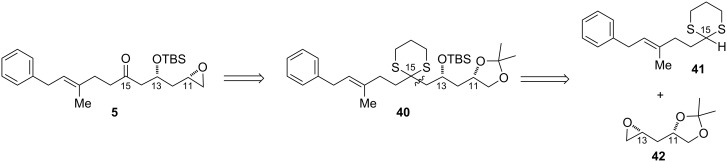
Initial retrosynthesis of the epoxide fragment by using dithiane coupling.

With respect to the synthesis of the proposed epoxide fragment **5**, we initially envisioned using the reaction of lithiated dithianes with epoxides (Figure 4) [45–49]. We reasoned that **41** could be expediently accessed from the corresponding aldehyde by making use of a Claisen rearrangement to set the geometry of the γ,δ-unsaturated double bond.

**Scheme 10:**
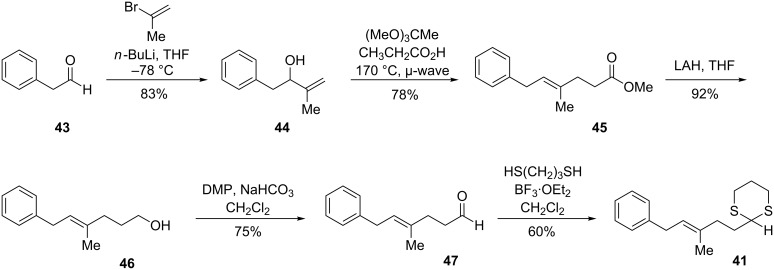
Synthesis of dithiane by Claisen rearrangement.

In the forward direction, the allylic alcohol **44** was obtained from reaction of the alkenyllithium reagent derived from 2-bromopropene with phenylacetaldehyde (Scheme 10). In our hands, the organolithium afforded significantly higher and more reproducible yields than either the commercially available Grignard reagent or the organocerium. Johnson–Claisen rearrangement [50] of **44** proceeded smoothly to give the γ,δ-unsaturated ester **45**. Conducting the reaction without added solvent in a microwave reactor at 170 °C allowed the reaction to proceed in just 30 minutes; a significant improvement over heating the reaction mixture in toluene under reflux, which typically required 48 hours to obtain a comparable yield. Reduction and oxidation afforded the aldehyde **47**, which could then be converted to dithiane **41**.

**Scheme 11:**
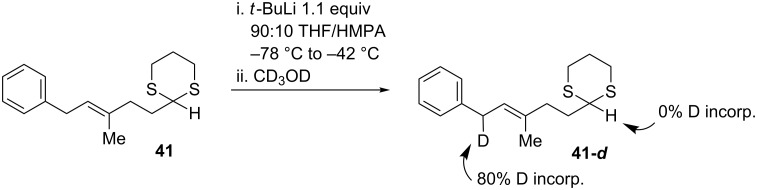
Deuterium labeling reveals that the allylic/benzylic site is most acidic.

Unfortunately, attempts to couple **41** with an oxygenated epoxide fragment under a variety of conditions reported by Smith for lithiation and electrophilic trapping [51] were unsuccessful. We suspected that the lithiated dithiane was not being generated and decided to investigate this step of the reaction independently of reaction with the epoxide electrophile. To this end, **41** was treated with *tert*-butyllithium at –78 °C in a 90:10 THF/HMPA mixture, referred to as the method of first choice for lithiation of complex dithianes (Scheme 11). Following warming to –42 °C, the reaction was quenched with deuterated methanol. Analysis of the product by ^1^H NMR revealed that no deuterium incorporation (to the sensitivity of integration) had occurred at the desired dithiane site, while approximately 80% deuterium incorporation had occurred at the allylic/benzylic site.

These results indicated that the presence of the trisubstituted C18–C19 olefin would interfere with dithiane coupling. However, given the suitability of the Claisen rearrangement for formation of this bond, we wished to preserve that transformation. Accordingly, an alternate route that would capitalize on the electrophilic nature of aldehyde **47** to form the bond corresponding to C14–C15 of ripostatin A instead was sought.

### Oxy-Michael approach to epoxide

**Scheme 12:**
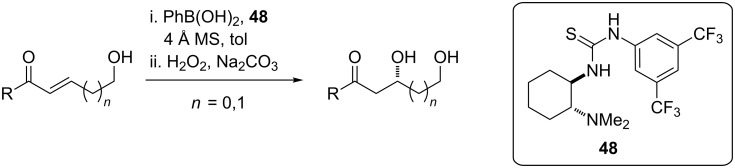
Oxy-Michael addition to δ-hydroxy-α,β-enones.

We were intrigued by a recent report by Falck describing an organocatalytic oxy-Michael addition to achiral δ-hydroxy-α,β-enones (Scheme 12) [52]. The hydroxy group is delivered in a directed fashion from the boronic acid hemiester generated in situ from the substrate and phenylboronic acid. It is proposed that complexation of the tertiary nitrogen to boron and coordination of the carbonyl act in a push/pull fashion, simultaneously enhancing the nucleophilicity of the boronate oxygen as well as imposing a chiral environment around the enone. Aliphatic enones react more sluggishly in this transformation; however, 3,4,5-trimethoxyphenylboronic acid may be used as a more efficient nucleophilic partner to circumvent this limitation.

**Figure 5:**
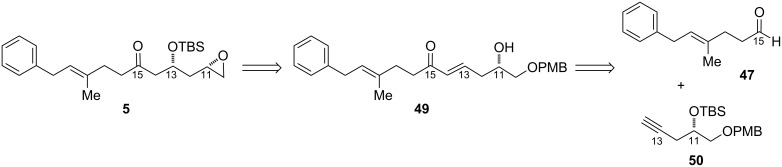
Revised retrosynthesis of epoxide **5**.

Application of this transformation to the ripostatin A epoxide fragment **5** allows installation of the C13 hydroxy group via conjugate addition to the δ-hydroxy-α,β-enone **49** (Figure 5). Although the diastereoselectivity of this reaction using substrates with a chiral center at the hydroxy group had not been investigated in the published study, substitution at the carbinol position was reportedly well tolerated for the reaction using γ-hydroxy-α,β-enones. Although the presence of chirality at the δ-position allows for diastereoselective intramolecular oxy-Michael addition of hemiacetal-derived alkoxides into α,β-unsaturated esters, the extension of this reaction to ketones was not successful [53]. In turn, we intended to prepare **49** by hydrometalation of **50** and addition into aldehyde **47**.

**Scheme 13:**
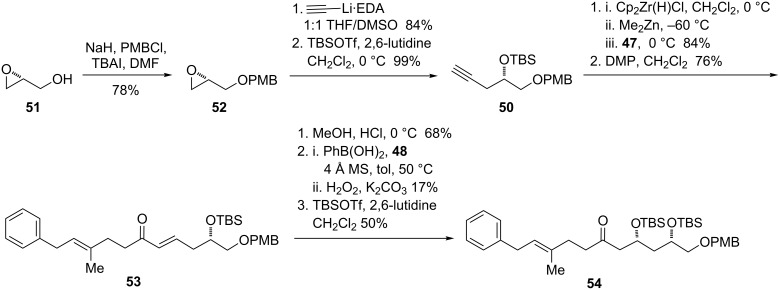
Synthesis of functionalized ketone by oxy-Michael addition.

To this end, (*R*)-glycidol was protected as the *para*-methoxybenzoate ether to give the PMB glycidol **52** (Scheme 13), in which the configuration is now assigned as (*S*). Opening the epoxide of **52** with the ethylenediamine complex of lithium acetylide in a 1:1 THF/DMSO solvent mixture at 0 °C allowed the terminal alkyne to be accessed in 84% yield without rearrangement to the internal alkyne. Silyl protection afforded alkyne **50**, which was prone to decomposition upon extended storage, even at −20 °C.

Hydrozirconation of alkyne **50** with Schwartz’s reagent [54–55] was followed by transmetallation to zinc and nonselective addition into aldehyde **47**. Oxidation of the resulting allylic alcohol mixture afforded the enone **53**. Prior to the key oxy-Michael addition, it was necessary to remove the *tert*-butyldimethylsilyl protecting group. Use of TBAF under buffered (acetic acid) or unbuffered conditions proved sluggish and somewhat low-yielding, typically ~50%. Various other promoters led to decomposition (BF_3_·OEt_2_) or low conversion (CsF); however, a modest improvement in yield was noted with the use of 1% HCl in MeOH (with a small amount of THF to dissolve the starting material).

The oxy-Michael addition with chiral thiourea and phenylboronic acid proceeded to give a single diastereomer; however, after 48 h at 50 °C, only 17% of the *syn* diol was formed, and 56% of the starting material was re-isolated. Under the modified conditions for less reactive aliphatic aldehydes using 3,4,5-trimethoxyphenylboronic acid, we were unable to isolate the desired diol from the reaction mixture. Since the oxy-Michael substrate derived from **53** contains an existing stereocenter at the directing hydroxy group, we also attempted to carry out the reaction with diisopropylamine as a substitute for the thiourea catalyst. This modification afforded both the *syn* and *anti* diols in roughly a 1:1 ratio and a combined yield of 40–45%, albeit without recovery of starting material. The *syn* and *anti* diastereomers could be separated by repeated silica gel chromatography, and the desired *syn* diol was converted to the bis-silylated compound **54**.

However, attempted oxidative deprotection of the PMB ether of **54** with DDQ led to destruction of the material. It seems likely that this is again due to the presence of the allylic/benzylic site in the molecule, although no individual decomposition products could be identified. Although it is possible that further screening of deprotection conditions might have allowed us to move forward with this route, the modest yields and long reaction time of the oxy-Michael addition severely limited material throughput. In order to proceed with the synthesis, we needed a more robust route, with the following criteria: (1) no protecting groups requiring oxidative cleavage, and (2) introduction of the C13 hydroxy group at an early stage.

### Iodocyclization approaches to epoxide

**Figure 6:**
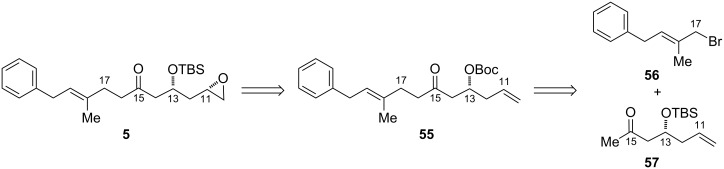
Retrosynthesis by using iodocylization to introduce the epoxide.

In the preparation of model epoxide **28**, iodocyclization was used to introduce oxygenation in a stereoselective fashion from a chiral homoallylic alcohol. Applying this disconnection, we hypothesized that we might be able to introduce the epoxide functional group of **5** by iodocyclization of the *tert*-butyl carbonate **55** (Figure 6). Although the additional double bond in this substrate presents a potential site for competing reaction pathways, we were encouraged by a report by Bartlett in which the iodocyclization of the *tert*-butyl carbonate derivative of 1,7-octadien-4-ol afforded exclusively the 6-membered carbonate derivative, with product arising from cyclization onto the more distant double bond not detected [56]. We set out to access **55** from deprotonation of methyl ketone **57** on the less hindered side and alkylation with bromide **56**.

**Scheme 14:**
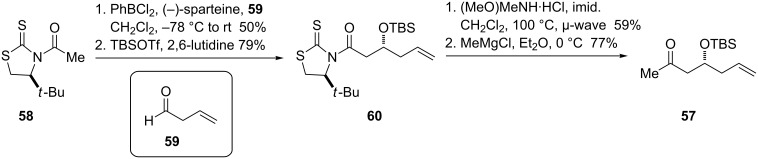
Synthesis of ketone **57** using thiazolidinethione chiral auxiliary.

The *E*-allylic bromide was prepared rapidly, albeit in modest yield, with an Appel reaction [57] of the known allylic alcohol [58]. To synthesize ketone **57**, we opted to utilize an asymmetric aldol reaction to set the stereochemistry of the β-hydroxy group. Since the report of Evans’s diastereoselective asymmetric aldol reaction using the boron enolates of *N*-acyloxazolidinones [59], numerous chiral-auxiliary-based methods have been developed for the synthesis of *syn*- or *anti*-propionate aldol units. However, many of these auxiliaries, including the Evans oxazolidinones, fail to give high stereoselectivities when employed in acetate aldol reactions [60]. Of the methods available, we selected Sammakia’s boron enolate-based strategy using the *N*-acetylthiazolidinethione **58** (Scheme 14) for its high reported diastereoselectivity with aliphatic aldehydes and its avoidance of toxic tin reagents [61].

The reaction of the brightly colored **58** with but-3-enal proceeded in moderate yield, with an initial diastereoselectivity around 10:1, with further enhancement following silyl protection and purification. Although the silylated compound **60** proved to be reluctant to form the Weinreb amide, microwave irradiation allowed this process to proceed on a reasonable time scale. Grignard addition to the Weinreb amide afforded ketone **57**.

**Figure 7:**
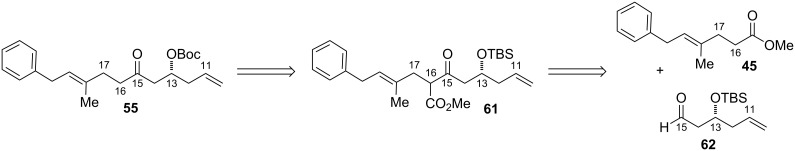
Retrosynthesis involving decarboxylation of a β-ketoester.

Unfortunately, attempts to unite ketone **57** and bromide **56** via alkylation were unsuccessful. Although deprotection at the less-substituted site of the methyl ketone using LDA was verified in a deuterium quench experiment, the alkylation did not proceed at temperatures from −78 °C to 0 °C. While the ketone was re-isolated cleanly following the reaction, the bromide was converted to a mixture of olefinic compounds. Faced with the difficulty of forming the C16−C17 bond by alkylation, we considered potential routes arising from retrosynthetic disconnection of the C15−C16 bond (Figure 7). It was recognized that reaction of the enolate of ester **45**, a compound previously synthesized in just two steps, and subsequent oxidation could give the β-ketoester **61**. Decarboxylation of this compound would provide rapid access to the key iodocyclization substrate **55**.

**Scheme 15:**
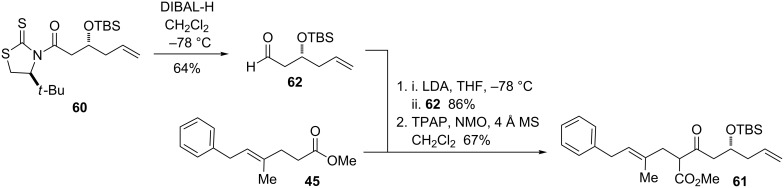
Synthesis of β-ketoester **61**.

Aldehyde **62** was prepared by reduction of the thiazolidinethione **60** with DIBAL-H (Scheme 15). Treatment of the ester with LDA, followed by trapping with the aldehyde, afforded the aldol adduct as a mixture of up to four possible diastereomers. This was then oxidized under Ley’s conditions [62] to the β-ketoester **61**, itself a mixture of two diastereomers.

**Scheme 16:**
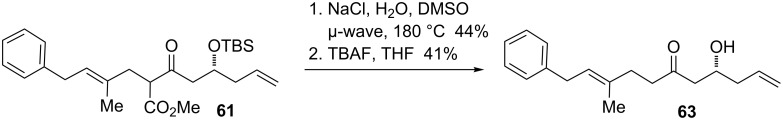
Decarboxylation of **61** under Krapcho conditions.

Initially, we attempted to induce decarboxylation of **61** by treatment with LiOH in a 1:1 water/THF mixture. No reaction was observed at room temperature, but heating to 70 °C resulted in elimination of the β-siloxy group. The Krapcho reaction offers an essentially neutral method for the decarboxylation of base- and acid-sensitive substrates [63]. Under these conditions (sodium chloride in wet DMSO at elevated temperatures) the desired decarboxylation reaction proceeded, although only in modest yield (Scheme 16). Reduction of the temperature from 180 °C to 120 °C led to much lower conversion, as expected, but did not improve mass recovery in the reaction. Somewhat more surprisingly, silyl deprotection with TBAF was also low yielding. Interestingly, when the order of these operations was reversed, the Krapcho conditions led to a complex product mixture, the major component of which appeared to be the dienone, formed by elimination and isomerization of the terminal olefin into conjugation.

**Scheme 17:**
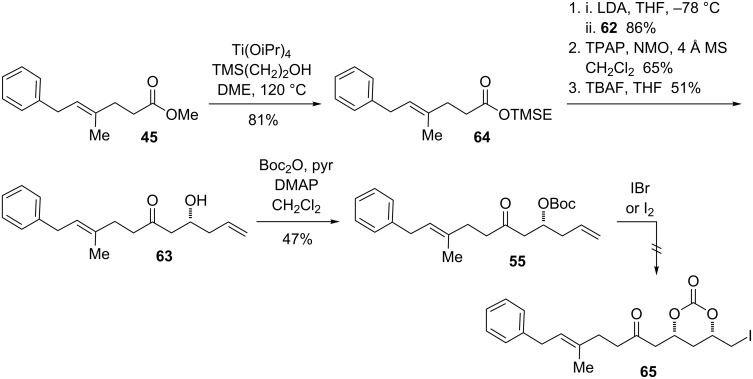
Improved synthesis of **63** and attempted iodocyclization.

We reasoned that the decarboxylation and silyl deprotection steps could be coupled into one operation by switching from the methyl ester to the 2-trimethylsilylethyl (TMSE) ester. Although there was concern that if the TBS group were removed first, the free hydroxy group would undergo elimination, it seemed likely that both deprotections would proceed at ambient temperature, which might circumvent this issue. Transesterification of methyl ester **45** to TMSE ester **64** proceeded in good yield, and following an analogous procedure for aldol reaction and oxidation the TMSE β-ketoester was obtained (Scheme 17). Treatment with an excess of TBAF in THF at room temperature overnight resulted in formation of the β-hydroxyketone **63**. Although the yield for this transformation remained moderate, it was higher than that obtained for either of the individual steps from the methyl series that it replaced.

The cleavage of TMSE β-ketoesters with TBAF·3H_2_O has been described in the literature as a chemoselective method for decarboxylation in the presence of other types of β-ketoesters [64]. Comparable yields for the decarboxylation to form **63** were obtained with this reagent as with the anhydrous solution, or when the reaction was run in DMF instead of THF. The use of TAS-F (tris(dimethylamino)sulfonium trifluoromethylsilicate) was clearly inferior, leading to incomplete conversion and elimination. With TBAF, partial elimination could sometimes be observed; however, this typically occurred in less than 10%. Given these results, it seems that the fluoride-mediated deprotection of TMSE β-ketoesters is deserving of further exploration and utilization as a method for the decarboxylation of sensitive synthetic intermediates.

Alcohol **63** was derivatized as the Boc carbonate, a reaction plagued by the formation of the carbonate arising from two molecules of **63**. The ratio of Boc derivative to symmetrical carbonates is dependent on the acidity of the alcohol and not necessarily improved by increasing the stoichiometry of Boc_2_O [65]. Disappointingly, treatment of **55** with IBr or I_2_ led to exhaustive decomposition of the material. Similarly, attempts to convert the homoallylic olefin of **55** into the epoxide via directed oxidation with VO(acac)_2_ and TBHP again resulted in an intractable mixture of products.

While investigations into installing the epoxide via iodocyclization were ultimately not fruitful, in the course of this route an expeditious and perhaps underappreciated disconnection was identified in the construction of the C15−C16 bond via a simple aldol reaction, followed by TBAF-promoted decarboxylation to remove the ester. We concluded that selective reaction of the terminal olefin in the presence of the trisubstituted olefin was not a feasible proposition. Therefore, a substrate with oxygenation present at C10 and C11 from an early stage was needed as well.

### Acetonide approach to epoxide

**Figure 8:**
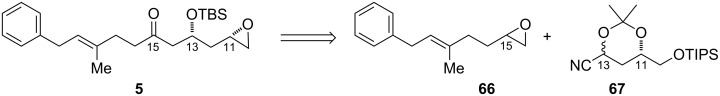
Retrosynthesis utilizing Rychnovsky’s cyanohydrin acetonide methodology.

To obtain the key C10−C11 epoxide in **5** in stereoselective fashion from displacement of a leaving group at C10, a means for the selective formation of a *syn*-1,3-diol at C11 and C13 is required. Rychnovsky has demonstrated that alkylation of 4-cyano-1,3-dioxanes (cyanohydrin acetonides) constitutes a practical and valuable approach to *syn*-1,3-diol synthesis [66]. The lithiated cyanohydrin acetonides react as nucleophiles with alkyl, allyl, and propargyl halides, as well as with epoxides. Although the alkylation itself is highly stereoselective in favor of the axial nitrile, the *syn*-1,3-diol stereochemistry is ultimately set in a subsequent reductive decyanation step. We planned to synthesize **5** by reaction of the cyanohydrin acetonide **67** with the epoxide electrophile **66** (Figure 8) [67].

**Scheme 18:**
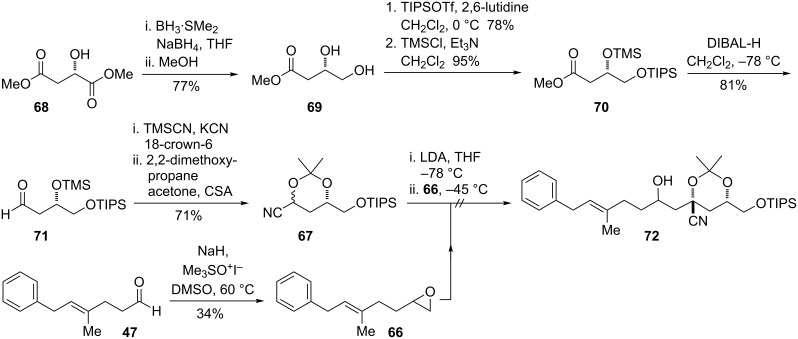
Synthesis of cyanohydrin acetonide and attempted alkylation with epoxide.

The dimethyl derivative of L-malic acid was chemoselectively reduced with borane-dimethylsulfide and sodium borohydride to afford diol **69** (Scheme 18) [68]. The primary alcohol was protected as the TIPS ether, and the secondary alcohol subsequently converted to the TMS ether. Reduction with DIBAL-H afforded the aldehyde **71** without over-reduction to the alcohol. Acetonide formation proceeded smoothly to give **67** as an inconsequential mixture of diastereomers. However, attemps to alkylate the lithiated anion of **67** with epoxide **66** led only to recovery of starting material. Interestingly, although alkylations of the acetonide are known to be stereoselective, protonation does not appear to be, as a *cis* and *trans* mixture was obtained from the attempted reaction of a single acetonide diastereomer.

**Scheme 19:**
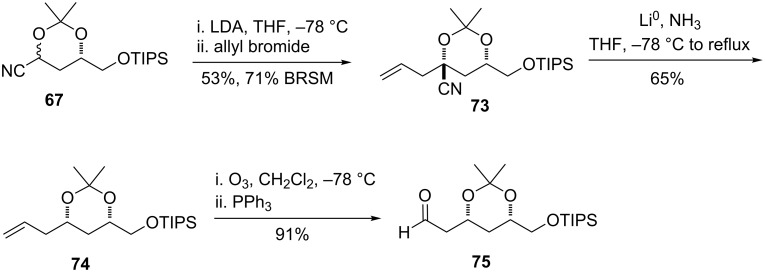
Allylation of acetonide and conversion to aldehyde.

In the course of investigating why **66** and **67** failed to react, attempts were made to trap the anion of **67** with a more reactive electrophile. Allyl bromide reacted rapidly, affording the product **73** as a single diastereomer (Scheme 19). The configuration of this compound, as well as subsequent compounds along this route, could be assigned as the 1,3-*syn* acetonide by analysis of the ^13^C chemical shifts of the acetonide methyl groups [69]. It was recognized that conversion of this olefin to the aldehyde would provide an ideal electrophile for a revised β-ketoester decarboxylation strategy. To this end, reduction of the nitrile proceeded with the expected selectivity; this arises from equilibration of the intermediate radical to the more stable axial radical. It was essential to allow the reaction to warm under reflux due to the insolubility of the starting material in liquid ammonia. Ozonolysis of the pendant olefin afforded aldehyde **75** in high yield.

**Scheme 20:**
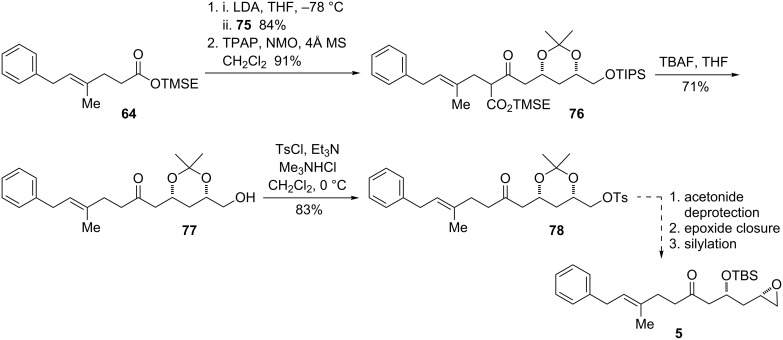
Synthesis of the epoxide precursor by an aldol−decarboxylation sequence.

Following the prior procedure, the lithium enolate of TMS ester **64** was reacted with aldehyde **75**, and the mixture of diastereomeric alcohols was oxidized to the β-ketoester **76** (Scheme 20). This substrate did not appear to be prone to elimination, and treatment of the β-ketoester with TBAF in THF provided the decarboxylated and deprotected alcohol **77**. The primary alcohol could be converted to the tosylate **78** in good yield with tosyl chloride, triethylamine, and trimethylamine hydrochloride as the catalyst.

The alcohol **77** and tosylate **78** contain all of the carbon atoms of epoxide fragment **5** in the correct oxidation state. The remaining steps required to access the epoxide consist of acetonide deprotection, displacement of the tosylate or another appropriate leaving group to obtain the terminal epoxide, and silyl protection.

## Conclusion

Nickel-catalyzed reductive coupling methodologies are an attractive fragment coupling strategy for the synthesis of complex natural products. The formation of sensitive skipped diene units in this context remains largely an unsolved problem for organic chemistry, but reactions for the rearrangement of vinylcyclopropanes present an intriguing avenue for exploration. To facilitate future studies in this vein, a cyclopropylenyne corresponding to the C1−C9 carbons of ripostatin A was synthesized in ten steps and up to 35% yield, and was shown to be a viable substrate in a nickel-catalyzed coupling reaction. The lack of complete regioselectivity in this transformation highlights that the factors governing selectivity in this reaction are more complex and more substrate dependent than initially anticipated.

Several strategies for synthesis of the C10−C26 epoxide fragment were examined, including dithiane linchpin coupling, oxy-Michael addition to an enone, and iodocyclization methods. Fluoride-promoted decarboxylation of TMSE esters was identified as a mild strategy enabling simple aldol reactions for the construction of the C15−C16 bond. Allylation and reductive decyanation of a lithiated cyanohydrin acetonide was used to set the C13 stereocenter. Additional efforts are needed to convert a fully oxygenated, ketone-containing triol into the critical epoxide fragment and to investigate the rearrangement of reductive coupling products.

## Supporting Information

File 1 Experimental procedures and characterization data for newly synthesized compounds.
