# Supplementary material for: A reductive coupling strategy towards ripostatin A
Source: Beilstein J Org Chem. 2013 Jul 31;9:1533–50. doi: 10.3762/bjoc.9.175 (PMC3740708; doi:10.3762/bjoc.9.175)
Supplement: File 1 — Experimental procedures and characterization data for newly synthesized compounds. [file Beilstein_J_Org_Chem-09-1533-s001.pdf]

Supporting Information

for

A reductive coupling strategy towards ripostatin A

Kristin D. Schleicher and Timothy F. Jamison*

Address: Department of Chemistry, Massachusetts Institute of Technology, 77 Massachusetts Ave., Cambridge, MA 02139, United States

Email: Timothy F. Jamison - tfj@mit.edu

* Corresponding author

Experimental procedures and characterization data for newly synthesized compounds

General Information. Unless otherwise noted, all reactions were performed under an oxygen-free atmosphere of argon with rigorous exclusion of moisture from reagents and glassware. Dichloromethane and toluene were either distilled from calcium hydride or purified via a SG Water USA solvent purification system. Tetrahydrofuran and diethyl ether were either distilled from a blue solution of sodium benzophenone ketyl or purified via a SG Water solvent purification system.

Analytical thin layer chromatography was performed using EM Science silica gel 60 F254 plates. The developed chromatogram was analyzed by UV lamp (254 nm) and ethanolic phosphomolybdic acid (PMA), potassium permanganate ($KMnO_4$), cerium ammonium nitrate (CAM), iodine, or vanillin. Liquid chromatography was performed

using a forced flow (flash chromatography) of the indicated solvent system on Silicycle silica gel (230–400 mesh).

^{1}H and ^{13}C spectra were recorded in $CDCl_3$ on a Varian Inova 500 MHz spectrometer. Chemical shifts in ^{1}H NMR spectra are reported in parts per million (ppm) on the δ scale from an internal standard of residual chloroform (7.27 ppm). Data are reported as follows: chemical shift, multiplicity (s = singlet, d = doublet, t = triplet, q = quartet, m = multiplet, app = apparent, and br = broad), coupling constants in hertz (Hz), and integration. Chemical shifts in ^{13}C NMR spectra are reported in ppm from the central peak of $CDCl_3$ (77.23 ppm). Infrared (IR) spectra were recorded on a Perkin–Elmer 2000 FTIR. High resolution mass spectra (HRMS) were obtained on a Bruker Daltonics APEXII 3 Fourier Transform Mass Spectrometer by Ms. Li Li of the Massachusetts Institute of Technology Department of Chemistry Instrumentation Facility, or on a Waters Qtof API US instrument by Dr. Norman Lee of the Boston University Chemical Instrumentation Center. Elemental analysis (EA) was performed by Atlantic Microlab, Inc. Specific rotations ($[\alpha]_D$) were measured on a Jasco 1010 polarimeter at 589 nm where applicable, and concentrations are reported in g/100 mL of the given solvent.

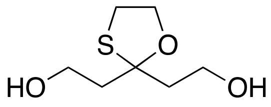

17

2,2′-(1,3-Oxathiolane-2,2-diyl)diethanol (17): Using a procedure analogous to one reported in the literature,[1] to a solution of diethyl 1,3-acetonedicarboxylate (14.5 mL, 80.0 mmol, 1.0 equiv) in CH_2Cl_2 (100 mL) was added 2-mercaptoethanol (9.8 mL, 140 mmol, 1.75 equiv) and $BF_3{\cdot}OEt_2$ (12.3 mL, 100 mmol, 1.25 equiv). The reaction was stirred overnight at rt. The reaction was diluted with Et_2O (160 mL) and quenched with H_2O (80 mL). The aqueous phase was separated, and the organics were washed with saturated aq. $NaHCO_3$ (50 mL) followed by brine (50 mL). The organic phase was then dried over $MgSO_4$, filtered, and concentrated in vacuo. Silica gel chromatography (2:1 hexanes/EtOAc) afforded the protected diester as a colorless liquid in greater than 90% purity, which was carried on to the next step (20.10 g).

A three-necked round-bottom flask equipped with stir bar and addition funnel was charged with LAH (5.82 g, 153 mmol, 2.0 equiv) and THF (300 mL). The reaction was cooled to 0 °C, and a solution of diester (20.10 g, 76.6 mmol, 1.0 equiv) in THF (50 mL) was added dropwise under stirring. The addition funnel was replaced with a rubber septum, and the reaction was stirred for 4 h, gradually warming to rt. The reaction was quenched with H_2O (6 mL, vigorous hydrogen gas evolution), followed by 2 M NaOH (6 mL), then H_2O (15 mL). The reaction was stirred an additional 30 min, then filtered through Celite, washing with EtOAc. The filtrate was concentrated in vacuo. Silica gel chromatography (95:5 to 90:10 CH_2Cl_2/MeOH) afforded the title compound (11.69 g, 82% over 2 steps), with ^{1}H NMR data in agreement with that reported in the literature.

^{1}H NMR (500 MHz, $CDCl_3$): δ 4.18 (t, *J* = 5.9 Hz, 2H); 3.79 (t, *J* = 5.8 Hz, 4H); 3.09 (t, *J* = 5.9 Hz, 2H); 3.03 (br, 2H); 2.15 (m, 4H).

^{13}C NMR (125 MHz, $CDCl_3$): δ 97.0, 70.7, 59.4, 43.1, 33.9.

IR (NaCl plate, thin film, cm^{-1}): 3362, 2949, 2883, 1472, 1438, 1351, 1268, 1214, 1045, 912, 880, 733, 647.

HRMS-ESI (*m*/*z*): $[M – H]^+$ calculated for $C_7H_{14}O_3S$, 177.0580; found, 177.0580.

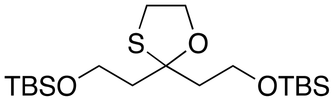

18

(((1,3-Oxathiolane-2,2-diyl)bis(ethane-2,1-diyl))bis(oxy))bis(*tert*-butyldimethylsilane) (18): Diol **17** (1.74 g, 9.76 mmol, 1.0 equiv) was dissolved in CH_2Cl_2 (100 mL). To this was added imidazole (2.66 g, 39.0 mmol, 4.0 equiv), followed by TSBCl (2.94 g, 19.5 mmol, 2.0 equiv). The solution became white and cloudy, and was stirred at rt overnight. After 16 h, the reaction was quenched with saturated aq. NH_4Cl. The organic phase was washed twice with brine, then dried with $MgSO_4$, filtered, and concentrated in vacuo. Silica gel chromatography (90:10 hexanes/EtOAc) afforded the title compound as a colorless liquid (3.50 g, 88%).

^{1}H NMR (500 MHz, $CDCl_3$): δ 4.11 (t, *J* = 5.9 Hz, 2H); 3.77 (m, 4H); 3.01 (t, *J* = 5.9 Hz, 2H); 2.11 (m, 4H); 0.90 (s, 18H); 0.06 (s, 12H).

^{13}C NMR (125 MHz, $CDCl_3$): δ 95.5, 70.4, 60.2, 44.1, 33.9, 26.2, 18.5, –5.1.

IR (NaCl plate, thin film, cm^{-1}): 2955, 2929, 2884, 2857, 1472, 1463, 1437, 1389, 1361, 1256, 1096, 1006, 939, 837, 775.

HRMS-ESI (*m*/*z*): $[M + Na]^+$ calculated for, $C_{19}H_{42}O_3SSi_2$, 429.2285; found, 429.2294.

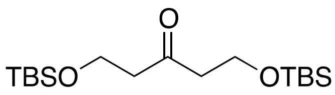

19

2,2,3,3,11,11,12,12-Octamethyl-4,10-dioxa-3,11-disilatridecan-7-one (19): In a 200 mL round bottom flask equipped with stir bar, compound **18** (2.84 g, 6.98 mmol, 1.0 equiv) in Et_2O (5 mL) was dissolved in 4:1 $MeCN/H_2O$ (50 mL). Calcium carbonate (1.40 g, 14.0 mmol, 2.0 equiv) was added, then $HgCl_2$ (4.17 g, 15.4 mmol, *Caution: Toxic!*), and the reaction was stirred at rt for 4 h. The mixture was filtered through a pad of diatomaceous earth, washing with Et_2O. The filtrate was washed with saturated aq. NH_4Cl and extracted with Et_2O. The combined organics were dried over $MgSO_4$, filtered, and concentrated in vacuo. Silica gel chromatography (90:10 hexanes/EtOAc) afforded the title compound as a colorless liquid (2.15 g, 89%).

^{1}H NMR (500 MHz, $CDCl_3$): δ 3.89 (t, *J* = 6.4 Hz, 4H); 2.66 (t, *J* = 6.4 Hz, 4H); 0.88 (s, 18H); 0.05 (s, 12H).

^{13}C NMR (125 MHz, $CDCl_3$): δ 209.2, 58.9, 46.7, 26.1, 18.4, –5.2.

IR (NaCl plate, thin film, cm^{-1}): 2956, 2930, 2885, 2858, 1716, 1472, 1464, 1389, 1361, 1256, 1102, 1006, 939, 836, 812, 777.

HRMS-ESI (*m*/*z*): $[M + Na]^+$ calculated for $C_{17}H_{38}O_3Si_2$, 369.2252; found, 369.2247.

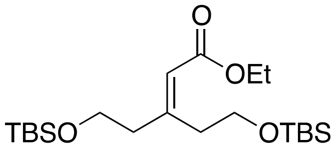

20

Ethyl 5-((*tert*-butyldimethylsilyl)oxy)-3-(2-((*tert*-butyldimethylsilyl)oxy)ethyl)pent-2-enoate (20): In a 100 mL flask with stir bar, *n*-BuLi (2.5 M in hexanes, 2.4 mL, 6.0 mmol, 1.5 equiv) was added to a solution of diisopropylamine (841 μL, 6.0 mmol, 1.5 equiv) in THF (30 mL) at 0 °C. The mixture was stirred for 10 minutes, then cooled

to –78 °C. Upon cooling, ethyl trimethylsilylacetate (1.10 mL, 6.0 mmol, 1.5 equiv) was added. The reaction was stirred for 10 minutes at –78 °C, then ketone **19** (1.39 g, 4.0 mmol, 1.0 equiv) was added slowly as a solution in THF (10 mL). The reaction was stirred at –78 °C for 2.5 h, then at –30 °C for 30 min. The mixture was quenched with sat. aq. NH_4Cl and extracted four times with EtOAc. The combined organics were dried with $MgSO_4$, filtered, and concentrated in vacuo. Silica gel chromatography (95:5 hexanes/EtOAc) afforded the title compound (1.57 g, 94%).

^{1}H NMR (500 MHz, $CDCl_3$): δ 5.72 (s, 1H); 4.14 (q, *J* = 7.1 Hz, 2H); 3.78 (t, *J* = 6.6 Hz, 2H); 3.75 (t, *J* = 6.7 Hz, 2H); 2.85 (t, *J* = 6.6 Hz, 2H); 2.43 (td, J_t = 6.7 Hz, J_d = 0.9 Hz, 2H); 1.27 (t, *J* = 7.1 Hz, 3H); 0.89 (s, 9H); 0.88 (s, 9H); 0.05 (s, 12H).

^{13}C NMR (125 MHz, $CDCl_3$): δ 166.4, 158.8, 118.3, 62.7, 61.6, 59.7, 42.9, 36.0, 26.1 (2 peaks), 18.5, 14.5, –5.1, –5.2.

IR (NaCl plate, thin film, cm^{-1}): 2956, 2930, 2886, 2858, 1717, 1645, 1473, 1388, 1362, 1256, 1189, 1146, 1098, 1039, 1006, 939, 836, 812, 776.

HRMS-ESI (*m*/*z*): $[M + Na]^+$ calculated for $C_{21}H_{44}O_4Si_2$, 439.2670; found, 439.2689.

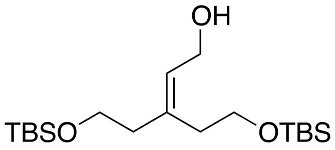

21

5-((*tert*-Butyldimethylsilyl)oxy)-3-(2-((*tert*-butyldimethylsilyl)oxy)ethyl)pent-2-en-1-ol (21): A solution of ester **20** (1.40 g, 3.36 mmol, 1.0 equiv) in CH_2Cl_2 (30 mL) was cooled to –78 °C. To this, DIBAL-H (1.0 M in CH_2Cl_2, 7.4 mL, 7.4 mmol, 2.2 equiv) was added dropwise. The reaction was stirred for 5 h at –78 °C. Rochelle's salt was added to quench, and the mixture was stirred for 45 minutes until phase separation occurred. The organic layer was separated, and the aqueous layer extracted with EtOAc. The combined organics were dried with $MgSO_4$, filtered, and concentrated in vacuo. Silica gel chromatography (80:20 hexanes/EtOAc) afforded the title compound (1.15 g, 92%).

^{1}H NMR (500 MHz, $CDCl_3$): δ 5.73 (t, *J* = 7.3 Hz, 1H); 4.05 (dd, *J* = 5.8, 7.2 Hz, 2H); 3.70 (m, 4H); 2.45 (t, *J* = 5.8 Hz, 1H); 2.38 (t, *J* = 5.9 Hz, 2H); 2.26 (t, *J* = 7.4 Hz, 1H); 0.90 (s, 9H); 0.89 (s, 9H); 0.07 (s, 6H); 0.05 (s, 6H).

^{13}C NMR (125 MHz, $CDCl_3$): δ 138.9, 128.2, 62.7, 61.1, 58.2, 39.8, 34.0, 26.2, 26.1, 18.5, –5.1, –5.3.

IR (NaCl plate, thin film, cm^{-1}): 3384, 2955, 2858, 1663, 1472, 1389, 1361, 1256, 1094, 1006, 921, 836, 812, 776, 664.

HRMS-ESI (*m*/*z*): $[M + Na]^+$ calculated for $C_{19}H_{42}O_3Si_2$, 397.2565; found, 397.2563.

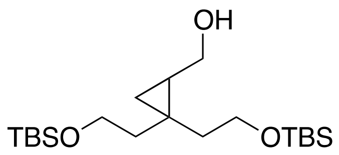

22

(2,2-bis(2-((*tert*-Butyldimethylsilyl)oxy)ethyl)cyclopropyl)methanol (22): To a solution of allylic alcohol **21** (1.13 g, 3.01 mmol, 1.0 equiv) in CH_2Cl_2 (30 mL) at 0 °C was added diethyl zinc (2.47 mL, 24.1 mmol, 8.0 equiv), followed by CH_2I_2 (970 μL, 12.0 mmol, 4.0 equiv). The reaction was stirred overnight, warming gradually to rt. The reaction was then quenched with saturated aq. NH_4Cl, upon which gas evolution was observed. The aqueous phase was separated and extracted with CH_2Cl_2, and the combined organics were washed with brine. The combined organics were dried over $MgSO_4$, filtered, and concentrated in vacuo. Silica gel chromatography (80:20 hexanes/EtOAc) afforded the title compound (1.08 g, 92%).

^1H NMR (500 MHz, $CDCl_3$): δ 3.95 (td, J_t = 10.7 Hz, J_d = 3.6 Hz, 1H); 3.85 (m, 2H); 3.71 (m, 3H); 3.25 (app t, 1H); 2.02 (dddd, *J* = 1.4, 7.1, 7.1, 14.2 Hz, 1H); 1.85 (dt, J_d = 15.1 Hz, J_t = 3.4 Hz, 1H); 1.52 (ddd, *J* = 5.2, 10.8, 15.6 Hz, 1H); 1.05 (m, 2H); 0.94 (s, 9H); 0.89 (s, 9H); 0.53 (dd, *J* = 4.7, 8.7 Hz, 1H); 0.12 (s, 6H); 0.05 (s, 6H); 0.04 (m, 1H).

^{13}C NMR (125 MHz, $CDCl_3$): δ 62.9, 61.8, 61.4, 39.8, 32.7, 27.4, 26.3, 26.1, 19.1, 18.8, 18.5, 14.8, -5.1 (2 peaks), –5.2, –5.3.

IR (NaCl plate, thin film, cm^{-1}): 3447, 3058, 2992, 2930, 2885, 2858, 1472, 1388, 1361, 1256, 1096, 1055, 1007, 938, 836, 775, 662.

HRMS-ESI (*m*/*z*): $[M + H]^+$ calculated for $C_{20}H_{44}O_3Si_2$, 389.2902; found, 389.2890.

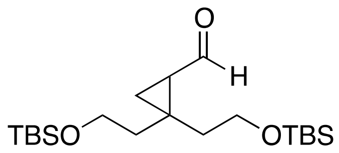

23

2,2-bis(2-((*tert*-Butyldimethylsilyl)oxy)ethyl)cyclopropanecarbaldehyde (23): To a solution of cyclopropyl alcohol **22** (1.82 g, 4.68 mmol, 1.0 equiv) in 1:1 CH_2Cl_2/DMSO (20 mL) was added triethylamine (2.61 mL, 18.7 mmol, 4.0 equiv). The mixture was cooled to 0 °C and SO_3·pyridine (1.49 g, 9.36 mmol, 2.0 equiv) was added. The cooling bath was removed and the reaction stirred for 15 min at rt. The reaction was quenched at 0 °C with saturated aq. NH_4Cl and extracted with CH_2Cl_2. The combined organics were dried with Na_2SO_4, filtered, and concentrated in vacuo. Silica gel chromatography (95:5 to 90:10 hexanes/EtOAc) afforded the title compound (1.72 g, 95%).

^{1}H NMR (500 MHz, $CDCl_3$): δ 9.42 (d, J = 5.1 Hz, 1H); 3.72 (t, J = 6.5 Hz, 2H); 3.69 (m, 2H); 1.90 (dt, J_d = 14.5 Hz, J_t = 6.2 Hz, 1H); 1.74 (m, 3H); 1.52 (dt, J_d = 14.2 Hz, J_t = 6.7 Hz, 1H); 1.34 (t, J = 5.1 Hz, 1H); 1.12 (dd, J = 4.8, 8.0 Hz, 1H); 0.90 (s, 9H); 0.89 (s, 9H); 0.06 (s, 6H); 0.05 (s, 6H).

^{13}C NMR (125 MHz, $CDCl_3$): δ 201.1, 61.4, 60.9, 40.0, 35.1, 33.0, 29.9, 26.2, 26.1, 21.2, 18.4, –5.2 (2 peaks).

IR (NaCl plate, thin film, cm^{-1}): 2955, 2930, 2886, 2856, 2737, 1706, 1472, 1463, 1389, 1361, 1256, 1104, 1031, 1005, 836, 811, 776.

HRMS-ESI (*m*/*z*): $[M – H]^+$ calculated for $C_{20}H_{42}O_3Si_2$, 385.2589; found, 385.2582.

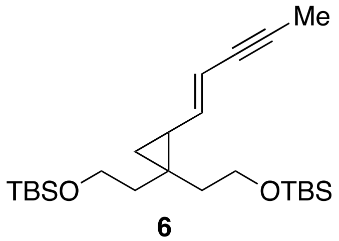

6

(*E*)-(((2-(Pent-1-en-3-yn-1-yl)cyclopropane-1,1-diyl)bis(ethane-2,1-diyl))bis(*tert*-butyldimethylsilane) (6): In a glovebox, a flame-dried, 50 mL round-bottomed flask was charged with $CrCl_2$ (1.68 g, 13.7 mmol, 9.0 equiv). The flask was removed from the glovebox and placed under Ar(g) atmosphere, and freshly distilled THF (10 mL) was

added. The flask was cooled to 0 °C and a solution of cyclopropyl aldehyde **23** (588 mg, 1.52 mmol, 1.0 equiv) and iodoform (1.80 g, 4.56 mmol, 3.0 equiv) in freshly distilled THF (14 mL) was added. The reaction changed in color from green to a reddish brown. The reaction was stirred overnight in the dark, gradually warming to rt. After 19 h, the reaction was quenched with water, upon which the color returned to green, and an extraction was performed with CH_2Cl_2. The combined organics were dried over $MgSO_4$, filtered, and concentrated in vacuo. Silica gel chromatography (hexanes to 95:5 hexanes/EtOAc) afforded the corresponding vinyl iodide (616 mg, 79%, 17:1 *E*/*Z*). The vinyl iodide is prone to *E/Z* isomerization and should be used directly, although storage for a period of several days in the refrigerator may be possible.

To a solution of vinyl iodide (405 mg, 0.794 mmol, 1.0 equiv) in THF (8 mL) was added diisopropylamine (0.56 mL, 4.0 mmol, 5.0 equiv), copper(I) iodide (15 mg, 0.079 mmol, 10 mol %), and tetrakis(triphenylphosphine)palladium(0) (46 mg, 0.040 mmol, 5 mol %). The mixture was cooled to –30 °C and condensed propyne (~ 0.8 mL) was added. The reaction was stirred at –30 °C for 27 h, and then quenched with saturated *aq*. NH_4Cl. The organic phase was separated, and the aqueous phase extracted with CH_2Cl_2. The combined organics were dried over $MgSO_4$, filtered, and concentrated in vacuo. Silica gel chromatography (97.5:2.5 hexanes/EtOAc) afforded the title compound as an orange oil (308 mg, 92%, 15:1 *E/Z*).

^{1}H NMR (500 MHz, $CDCl_3$): δ 5.83 (dd, J = 9.1, 15.6 Hz, 1H); 5.49 (dq, J_d = 15.6 Hz, J_q = 2.3 Hz, 1H); 3.69 (app td, 4H); 1.93 (d, J = 2.3 Hz, 1H); 1.56 (m, 3H); 1.45 (m, 1H); 1.35 (ddd, J = 5.4, 8.7, 8.7 Hz, 1H); 0.90 (s, 9H); 0.89 (s, 9H); 0.80 (dd, J = 4.8, 8.4 Hz, 1H); 0.50 (t, J = 5.1 Hz, 1H); 0.06 (m, 1H); 0.06 (s, 6H); 0.05 (s, 6H).

^{13}C NMR (125 MHz, $CDCl_3$): δ 143.4, 109.5, 84.6, 78.8, 61.7, 61.3, 40.7, 27.2, 26.2 (2 peaks), 23.9, 20.9, 18.5, –5.1 (2 peaks).

IR (NaCl plate, thin film, cm^{-1}): 2955, 2929, 2886, 2858, 1627, 1472, 1463, 1388, 1361, 1256, 1100, 1028, 1006, 949, 836, 812, 775, 661.

HRMS-ESI (*m*/*z*): $[M + H]^+$ calculated for $C_{24}H_{46}O_2Si_2$, 423.3019; found, 423.3111.

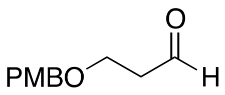

24

3-((4-Methoxybenzyl)oxy)propanal (24): As reported in the literature,[2] to a solution of 1,3-propanediol (60.12 g, 790.1 mmol, 5.0 equiv) in DMF (50 mL) at 0 °C was added sodium hydride (60% dispersion in mineral oil, 6.32 g, 158 mmol, 1.0 equiv). The mixture was stirred for 30 min at 0 °C, and a solution of PMBCl (21.4 mL, 158 mmol, 1.0 equiv) in DMF (40 mL) was then added. The reaction was stirred overnight, gradually warming from 0 °C to rt. The reaction mixture was quenched with saturated aq. NH_4Cl and extracted with EtOAc. The organic phase was washed with brine, dried over Na_2SO_4, filtered, and concentrated in vacuo. Silica gel chromatography (80:20 to 1:1 hexanes/EtOAc) afforded the mono-PMB-protected diol (20.56 g, 66%), which was carried forward to the next step.

To a solution of the mono-PMB-protected diol (20.56 g, 104.8 mmol, 1.0 equiv) in CH_2Cl_2 (52 mL) at 0 °C was added triethylamine (73.0 mL, 524 mmol, 5.0 equiv), DMSO (52 mL), and SO_3·pyridine (50.0 g, 314 mmol, 3.0 equiv). After stirring for 1.5 h at 0 °C, the reaction was quenched with pH 7 phosphate buffer solution (0.5 M, 800 mL). The aqueous phase was extracted with Et_2O (2 × 400 mL), then EtOAc (2 × 400 mL). The organics were washed once with brine, dried over Na_2SO_4, filtered, and concentrated in vacuo. Silica gel chromatography (80:20 to 2:1 hexanes/EtOAc) afforded the title compound (14.37 g, 71%), with spectroscopic data in agreement with literature values.[3]

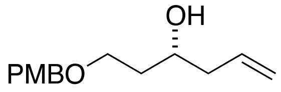

25

(*R*)-1-((4-Methoxybenzyl)oxy)hex-5-en-3-ol (25): In a glove box, two ampules of (+)-Ipc_2B(allyl) solution (1M in pentane, 50 mL, 50.0 mmol, 1.1 equiv) were transferred to a flame-dried 1 L round bottom flask equipped with stir bar. The flask was capped with a rubber septum, sealed with electrical tape, and removed from the glove box. Under an Ar(g) atmosphere, Et_2O (200 mL) was added. The flask was cooled to approximately −90 °C (liquid nitrogen/MeOH bath). To this was added a solution of aldehyde **24**

(8.74 g, 45.0 mmol, 1.0 equiv) in Et_2O (200 mL) via cannula. The reaction mixture was stirred for 1 h at −90 °C following addition, after which time 3 M NaOH (20 mL) and aq. H_2O_2 (10 mL) were added. The flask was equipped with a reflux condenser and heated at 35 °C for 2 h. The reaction mixture was allowed to return to rt, and water (400 mL) was added. The organic phase was separated, and the aqueous phase extracted with Et_2O (2 × 400 mL). The combined organics were dried over $MgSO_4$, filtered, and concentrated in vacuo. Silica gel chromatography (80:20 to 2:1 hexanes/EtOAc) afforded the title compound as a colorless liquid (9.45 g, 89%, 89% ee), with spectroscopic data in agreement with literature values.[4]

^{1}H NMR (500 MHz, $CDCl_3$): δ 7.26 (d, J = 8.5 Hz, 2H); 6.88 (d, J = 8.7 Hz, 2H); 5.84 (dddd, J = 7.1, 7.1, 10.3, 17.3 Hz, 1H); 5.10 (m, 2H); 4.46 (s, 2H); 3.87 (m, 1H); 3.81 (s, 3H); 3.70 (dt, J_d = 9.3 Hz, J_t = 5.3 Hz, 1H); 3.62 (m, 1H); 2.93 (d, J = 2.8 Hz, 1H); 2.25 (m, 2H); 1.76 (m, 2H).

^{13}C NMR (125 MHz, $CDCl_3$): δ 159.4, 135.1, 129.5, 117.7, 114.0, 73.2, 70.7, 68.9, 55.5, 42.1, 36.0.

IR (NaCl plate, thin film, cm^{-1}): 3439, 3075, 3001, 2936, 2862, 1641, 1613, 1586, 1514, 1465, 1442, 1364, 1303, 1249, 1174, 1091, 1035, 915, 822, 757, 733.

$[\alpha]_D$ = +4.6 (c = 0.60, $CHCl_3$, 23 °C).

Enantiomeric excess was established by HPLC analysis (Chiracel OD-H, 1.0 mL/min, 99.0:1.0 hexanes/iPrOH (t = 0.0 to 10.0 min) to 98.5:1.5 hexanes/iPrOH (t = 10.0 min to 25.0 min), t_R[minor] = 22.8 min, t_R[major] = 23.9 min).

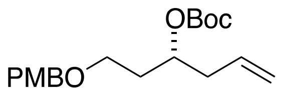

26

(*R*)-*tert*-Butyl (1-((4-methoxybenzyl)oxy)hex-5-en-3-yl carbonate (26): NaHMDS (6.02 g, 32.8 mmol, 1.3 equiv) was dissolved in THF (33 mL). In a separate flask, alcohol **25** (5.97 g, 25.3 mmol, 1.0 equiv) was dissolved in THF (75 mL) and cooled to 0 °C, to which the solution of NaHMDS was added via cannula. The mixture was stirred for 30 min at 0 °C. Boc anhydride (7.55 mL, 32.8 mmol, 1.3 equiv) was added. The cooling bath was removed and the reaction stirred for 30 min, warming to rt. The

reaction was quenched with brine and extracted with 1:1 hexanes/Et_2O. The combined organics were dried over $MgSO_4$, filtered, and concentrated in vacuo. Silica gel chromatography (90:10 hexanes/EtOAc) afforded the title compound as a colorless oil (7.09 g, 83%), with ^{1}H NMR data in agreement with literature values.[5]

^{1}H NMR (500 MHz, $CDCl_3$): δ 7.27 (d, *J* = 8.4 Hz, 2H); 6.88 (d, *J* = 8.4 Hz, 2H); 5.79 (dddd, *J* = 7.1, 7.1, 10.2, 17.2 Hz, 1H); 5.09 (m, 2H); 4.89 (pentet, *J* = 6.3 Hz, 1H); 4.42 (s, 2H); 3.81 (s, 3H); 3.50 (m, 2H); 2.37 (t, *J* = 6.8 Hz, 2H); 1.91 (d, *J* = 6.6 Hz, 1H); 1.87 (d, *J* = 6.6 Hz, 1H); 1.48 (s, 9H).

^{13}C NMR (125 MHz, $CDCl_3$): δ 159.3, 153.4, 133.6, 130.6, 129.5, 118.2, 114.0, 82.0, 74.1, 73.0, 66.4, 55.5, 39.2, 34.1, 28.0.

IR (NaCl plate, thin film, cm^{-1}): 2980, 2935, 2863, 1737, 1643, 1614, 1587, 1514, 1459, 1368, 1279, 1171, 1095, 1037, 920, 822, 755.

EA: Calculated for $C_{19}H_{28}O_5$: C, 67.83; H, 8.39. Found: C, 67.87; H, 8.37.

$[\alpha]_D$ = –27.0 (*c* = 1.11, $CHCl_3$, 24 °C).

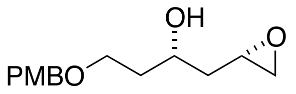

27

(*S*)-4-((4-Methoxybenxyl)oxy)-1-((*S*)-oxiran-2-yl)butan-2-ol (27): A round bottom flask equipped with stir bar and addition funnel was charged with carbonate **26** (2.02 g, 6.0 mmol, 1.0 equiv) and toluene (30 mL). The flask was cooled to –78 °C. A solution of IBr (1.0 M in CH_2Cl_2, 1.86 g, 9.0 mmol, 1.5 equiv) was added dropwise, and the reaction was stirred for 2 h following addition. The reaction was quenched with 1:1 saturated aq. $NaHCO_3$/$Na_2S_2O_3$ (100 mL) and extracted three times with Et_2O. The combined organics were dried over $MgSO_4$, filtered, and concentrated in vacuo. Silica gel chromatography (1:1 hexanes/EtOAc) afforded the iodocarbonate (1.96 g, 80%).

Iodocarbonate (1.95 g, 4.8 mmol, 1.0 equiv) was dissolved in MeOH (32 mL) and cooled to 0 °C. Potassium carbonate (1.99 g, 14.4 mmol, 3.0 equiv) was added, and the reaction was stirred overnight, gradually warming to rt. The reaction was quenched with water and extracted with CH_2Cl_2. The combined organics were dried over $MgSO_4$, filtered, and concentrated in vacuo. Silica gel chromatography (1:1 hexanes/EtOAc)

afforded the title compound (670 mg, 55%, > 20:1 dr), with spectroscopic data in agreement with those reported in the literature.[4]

^{1}H NMR (500 MHz, $CDCl_3$): δ 7.25 (d, *J* = 8.7 Hz, 2H); 6.88 (d, *J* = 8.7 Hz, 2H); 4.46 (s, 2H); 4.06 (dddd, *J* = 3.0, 4.4, 7.5, 8.9 Hz, 1H); 3.81 (s, 3H); 3.71 (ddd, *J* = 4.5, 5.7, 9.4 Hz, 1H); 3.64 (ddd, *J* = 4.2, 8.4, 9.4 Hz, 1H); 3.24 (br, 1H); 3.10 (m, 1H); 2.77 (m, 1H); 2.51 (dd, *J* = 2.7, 5.0 Hz, 1H); 1.85 (m, 1H); 1.75 (m, 2H); 1.67 (m, 1H).

^{13}C NMR (125 MHz, $CDCl_3$): δ 159.5, 130.1, 114.0, 73.2, 69.8, 68.8, 55.5, 50.2, 46.8, 40.0, 36.5.

IR (NaCl plate, thin film, cm^{-1}): 3457, 3045, 2998, 2938, 2863, 1613, 1586, 1514, 1465, 1442, 1421, 1362, 1303, 1249, 1175, 1092, 1034, 914, 824, 734.

$[\alpha]_D$ = +4.7 (*c* = 0.82, $CHCl_3$, 24 °C).

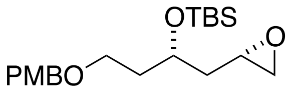

28

***tert*-Butyl(((*S*)-4-((4-methoxybenzyl)oxy)-1-((*S*)-oxiran-2-yl)butan-2-yl)oxy)dimethylsilane (28):** To a solution of epoxy alcohol **27** (1.15 g, 4.55 mmol, 1.0 equiv) in THF (40 mL) was added pyridine (1.84 mL, 22.8 mmol, 5.0 equiv) and silver(I) nitrate (1.16 g, 6.82 mmol, 1.5 equiv). The mixture was stirred for 10 min at rt, and then TBSCl (1.20 g, 7.96 mmol, 1.75 equiv) was added. The reaction was stirred overnight, then filtered to remove the precipitated silver chloride. The filtrate was poured into H_2O (100 mL) and extracted with Et_2O. The combined organics were dried over $MgSO_4$, filtered, and concentrated in vacuo. Silica gel chromatography (90:10 to 80:20 hexanes/EtOAc) afforded the title compound as a colorless oil (1.19 g, 71%).

^{1}H NMR (500 MHz, $CDCl_3$): δ 7.26 (d, *J* = 8.6 Hz, 2H); 6.88 (d, *J* = 8.6 Hz, 2H); 4.45 (d, *J* = 11.4 Hz, 1H); 4.39 (d, *J* = 11.4 Hz, 1H); 4.07 (dq, J_q = 5.4 Hz, J_d = 6.7 Hz, 1H); 3.81 (s, 3H); 3.53 (m, 2H); 3.05 (m, 1H); 2.75 (m, 1H); 2.44 (dd, *J* = 2.7, 5.1 Hz, 1H); 1.86 (m, 2H); 1.68 (t, *J* = 5.6 Hz, 2H); 0.89 (s, 9H); 0.07 (s, 3H); 0.06 (s, 3H).

^{13}C NMR (125 MHz, $CDCl_3$): δ 159.3, 130.8, 129.5, 114.0, 72.9, 67.6, 66.8, 55.5, 49.5, 47.0, 40.6, 37.3, 26.0, 18.2, –4.3, –4.5.

IR (NaCl plate, thin film, cm^{-1}): 3042, 2997, 2954, 2929, 2857, 1613, 1587, 1514, 1472, 1464, 1361, 1302, 1250, 1173, 1096, 1039, 1006, 837, 776.
EA: Calculated for $C_{20}H_{34}O_4Si$: C, 65.53; H, 9.35. Found: C, 65.62; H, 9.36.
$[\alpha]_D$ = –14.4 (*c* = 1.45, $CHCl_3$, 23 °C).

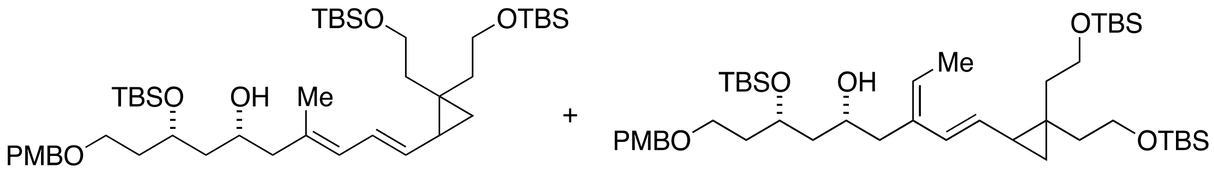

29 30

Reductive coupling products (29 and 30): To a 10 mL round-bottom flask containing stir bar, enyne **6** (137.5 mg, 0.325 mmol, 1.6 equiv), and epoxide **28** (74.5 mg, 0.203 mmol, 1.0 equiv) in the glove box were added $Ni(cod)_2$ (11.7 mg, 0.043 mmol, 20 mol %) and tributylphosphine (21 μL, 0.085 mmol, 40 mol %). Outside the glove box, under Ar(g), triethylborane (123 μL, 0.852 mmol, 4.0 equiv) was added. The reaction was stirred for 72 h at rt, then diluted with EtOAc (1 mL) and stirred open to the air for several hours. The reaction mixture was concentrated in vacuo. Silica gel chromatography (95:5 to 90:10 to 80:20 hexanes/EtOAc) afforded the product as a mixture of the desired reductive coupling product and its regioisomer (117.9 mg, 73%, 3:1). Further chromatography (85:15 hexanes/EtOAc) provided a clean sample of the major (desired) regioisomer **29** for analysis.
*Major regioisomer (***29***):*
^{1}H NMR (500 MHz, $CDCl_3$): 7.25 (d, *J* = 8.6 Hz, 2H); 6.88 (d, *J* = 8.6 Hz, 2H); 6.33 (dd, *J* = 11.0, 14.8 Hz, 1H); 5.86 (d, *J* = 11.1 Hz, 1H); 5.41 (dd, *J* = 9.1, 14.8 Hz, 1H); 4.44 (d, *J* = 11.5 Hz, 1H); 4.40 (d, *J* = 11.5 Hz, 1H); 4.07 (pentet, *J* = 6.1 Hz, 1H); 3.88 (m, 1H); 3.82 (s, 3H); 3.71 (m, 4H); 3.51 (t, *J* = 6.7 Hz, 2H); 2.15 (d, *J* = 6.6 Hz, 2H); 2.63-2.58 (two d, *J* = 1.8 Hz, 1H); 1.83 (m, 2H); 1.76 (s, 3H); 1.62-1.44 (m, 4H); 1.35 (m, 1H); 0.90 (2 singlets, 18H); 0.89 (s, 9H); 0.77 (dd, *J* = 5.1, 8.1 Hz, 1H); 0.45 (m, 1H); 0.10 (s, 3H); 0.09 (s, 3H); 0.06 (buried, 1H); 0.06 (s, 6H); 0.05 (s, 6H).
^{13}C NMR (125 MHz, $CDCl_3$): 159.3, 133.2, 132.0, 130.7, 129.5, 128.1, 126.7, 113.7, 72.9, 69.6, 67.8, 66.7, 61.8, 61.4, 55.5, 48.7, 43.8, 40.8, 37.6, 34.8, 27.2, 26.2 (2

peaks), 26.1, 23.2 (2 peaks), 20.6, 18.5 (2 peaks), 18.2, 17.0, –4.2, –4.3, –5.0, –5.1 (2 peaks).

IR (NaCl plate, thin film, cm^{-1}): 3451, 2929, 2857, 1613, 1514, 1472, 1361, 1251, 1173, 1098, 836, 775, 663.

HRMS-ESI (m/z): $[M + H]^+$ calculated for $C_{44}H_{82}O_6Si_3$, 813.5317; found, 813.5310.

$[\alpha]_D$ = –2.5 (c = 0.28, $CHCl_3$, 24 °C).

*Minor regioisomer (**30**):*

^{1}H NMR (500 MHz, $CDCl_3$, diagnostic peaks): 6.42 (dd, J = 6.7, 15.7 Hz, 1H); 5.53 (dd, J = 8.7, 15.9 Hz, 1H); 5.33 (app q, 1H).

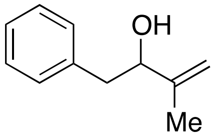

44

3-Methyl-1-phenylbut-3-en-2-ol (44): A solution of 2-bromopropene (5.77 mL, 65.0 mmol, 1.3 equiv) was dissolved in THF (160 mL) and cooled to –78 °C. To this was added *n*-butyllithium (titrated, 2.34 M in hexanes, 32.1 mL, 75.0 mmol, 1.5 equiv) slowly via syringe. The mixture was stirred for 15 min at –78 °C. Phenylacetaldehyde (90%, 6.50 mL, 50.0 mmol, 1.0 equiv) was added as a solution in THF (80 mL) via cannula. The reaction was stirred for an additional 30 min at –78 °C, then moved to an ice bath at 0 °C and promptly quenched with 1:1 saturated aq. NH_4Cl/H_2O, then extracted with EtOAc. The combined organics were dried over $MgSO_4$, filtered, and concentrated in vacuo. Silica gel chromatography (90:10 to 85:15 hexanes/EtOAc) afforded the title compound as a yellow liquid (6.73 g, 83%), with spectroscopic data in agreement with literature values.[6]

^{1}H NMR (500 MHz, $CDCl_3$): δ 7.33 (m, 2H); 7.25 (m, 3H); 4.97 (m, J = 0.9 Hz, 1H); 4.88 (m, J = 1.5 Hz, 1H); 4.30 (m, 1H); 2.93 (dd, J = 4.4, 13.7 Hz, 1H); 2.78 (dd, J = 8.7, 13.7, 1H); 1.83 (s, 3H); 1.64 (d, J = 3.4, 1H).

^{13}C NMR (125 MHz, $CDCl_3$): δ 146.9, 138.5, 129.5, 128.6, 126.7, 111.5, 76.7, 42.3, 18.3.

IR (NaCl plate, thin film, cm^{-1}): 3412, 3064, 3029, 2921, 1709, 1650, 1604, 1496, 1454, 1373, 1080, 1030, 904, 736, 699.
HRMS-ESI (m/z): $[M – H]^+$ calculated for $C_{11}H_{14}O$, 161.0961; found, 161.0965.

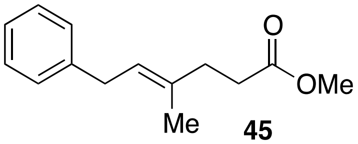

45

(*E*)-Methyl 4-methyl-6-phenylhex-4-enoate (45): Alcohol **44** (8.97 g, 55.3 mmol, 1.0 equiv), trimethyl orthoacetate (28.1 mL, 221 mmol, 4.0 equiv), and propionic acid (206 μL, 2.77 mmol, 0.05 equiv) were combined, and the mixture was distributed evenly into four 20 mL Biotage microwave vials. Each vial was irradiated at 170 °C for 30 min with stirring using a Biotage Initiator Eight microwave reactor. The contents of all four vials were combined and concentrated in vacuo. Silica gel chromatography (95:5 to 90:10 hexanes/EtOAc) afforded the title compound as a pale yellow liquid (9.40 g, 78%).
^{1}H NMR (500 MHz, $CDCl_3$): δ 7.29 (m, 2H); 7.18 (m, 3H); 5.39 (tq, J_t = 7.4 Hz, J_q = 1.3 Hz, 1H); 3.65 (s, 3H); 3.36 (d, J = 7.3 Hz, 2H); 2.47 (m, 2H); 2.38 (app t, 2H); 1.74 (s, 3H).
^{13}C NMR (125 MHz, $CDCl_3$): δ 174.0, 141.6, 134.7, 128.6, 128.5, 126.0, 124.1, 51.7, 34.9, 34.4, 33.1, 16.3.
IR (NaCl plate, thin film, cm^{-1}): 3062, 3027, 2951, 1740, 1603, 1494, 1436, 1347, 1295, 1258, 1196, 1162, 1090, 1072, 1030, 865, 742, 699.
HRMS-ESI (*m*/*z*): $[M + Na]^+$ calculated for $C_{14}H_{18}O_2$, 241.1199; found, 241.1201.

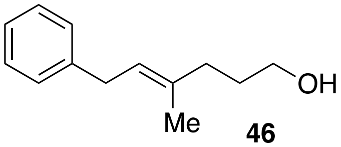

46

(*E*)-4-Methyl-6-phenylhex-4-en-1-ol (46): A solution of methyl ester **45** (9.25 g, 42.4 mmol, 1.0 equiv) in THF (200 mL) was cooled to 0 °C. Lithium aluminum hydride (1.61 g, 42.4 mmol, 1.0 equiv) was added in small portions over a period of 15 minutes, and the reaction was stirred for 3 h, gradually warming to rt. The reaction was carefully

quenched with water (1.6 mL), then 2 M NaOH (1.6 mL), then water (4.8 mL), and was stirred for 30 min. The mixture was filtered through Celite, washing with Et_2O. The filtrate was concentrated in vacuo. Silica gel chromatography (80:20 hexanes/EtOAc) afforded the title compound as a colorless liquid (7.45 g, 92%), with spectroscopic data in agreement with the literature values.[7]

^{1}H NMR (500 MHz, $CDCl_3$): δ 7.29 (m, 2H); 7.19 (m, 3H); 5.40 (tq, J_t = 7.3 Hz, J_q = 1.1 Hz, 1H); 3.66 (app q, 2H); 3.37 (d, *J* = 7.3 Hz, 2H); 2.14 (t, *J* = 7.5 Hz, 2H); 1.75 (s, 3H); 1.73 (m, 2H).

^{13}C NMR (125 MHz, $CDCl_3$): δ 141.8, 136.0, 128.6, 128.5, 126.0, 123.7, 63.0, 36.2, 34.4, 31.0, 16.3.

IR (NaCl plate, thin film, cm^{-1}): 3323, 3084, 3062, 3027, 2939, 2876, 1603, 1494, 1452, 1382, 1057, 916, 742, 698.

HRMS-ESI (*m*/*z*): $[M - H]^+$ calculated for $C_{13}H_{18}O$, 189.1274; found, 189.1276.

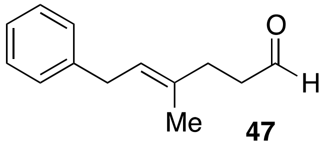

47

(*E*)-4-Methyl-6-phenylhex-4-enal (47): To a solution of alcohol **46** (1.35 g, 7.10 mmol, 1.0 equiv) in CH_2Cl_2 (35 mL) was added $NaHCO_3$ (1.79 g, 21.3 mmol, 3.0 equiv), then Dess–Martin periodinane (3.31 g, 7.80 mmol, 1.1 equiv). The reaction was stirred at rt for 3 h, then quenched with the addition of 1:1 saturated aq. $NaHCO_3/Na_2S_2O_3$ (40 mL). The organic phase was separated, and the aqueous phase extracted with EtOAc. The combined organics were dried over $MgSO_4$, filtered, and concentrated in vacuo. Silica gel chromatography (90:10 hexanes/EtOAc) afforded the title compound as a pale yellow liquid (999 mg, 75%), with spectroscopic data in agreement with literature values.[6]

^{1}H NMR (500 MHz, $CDCl_3$): δ 9.78 (t, *J* = 1.8 Hz, 1H); 7.29 (m, 2H); 7.17 (m, 3H); 5.39 (tq, J_t = 7.3 Hz, J_q = 1.3 Hz, 1H); 3.37 (d, *J* = 7.3 Hz, 2H); 2.58 (app td, 2H); 2.39 (t, *J* = 7.5 Hz, 2H); 1.75 (s, 3H).

^{13}C NMR (125 MHz, $CDCl_3$): δ 202.6, 141.4, 134.4, 128.6, 128.5, 126.1, 124.3, 42.3, 34.4, 32.0, 16.5.

IR (NaCl plate, thin film, cm^{-1}): 3084, 3062, 3027, 2914, 2828, 2722, 1725, 1603, 1494, 1453, 1410, 1388, 1074, 1030, 743, 699.

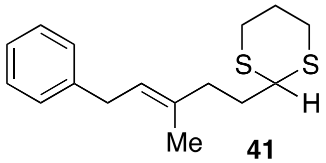

41

(*E*)-2-(3-Methyl-5-phenylpent-3-en-1-yl)-1,3-dithiane (41): To a solution of aldehyde **47** (565 mg, 3.00 mmol, 1.0 equiv) in CH_2Cl_2 (20 mL) was added 1,3-propanedithiol (602 μL, 6.00 mmol, 2.0 equiv). $BF_3{\cdot}OEt_2$ (370 μL, 3.00 mmol, 1.0 equiv) was added slowly, and the reaction was stirred for 2 h at rt. The reaction was then quenched with 10 wt % aq. NaOH (50 mL) and extracted with Et_2O. The combined organics were dried over Na_2SO_4, filtered, and concentrated in vacuo. Silica gel chromatography (95:5 hexanes/EtOAc) afforded the title compound as a pale yellow oil (501 mg, 60%).

^{1}H NMR (500 MHz, $CDCl_3$): δ 7.29 (m, 2H); 7.19 (m, 3H); 5.43 (tq, J_t = 7.4 Hz, J_q = 1.3 Hz, 1H); 3.99 (t, *J* = 6.9 Hz, 1H); 3.37 (d, *J* = 7.4 Hz, 2H); 2.83 (m, 2H); 2.82 (d, *J* = 3.7 Hz, 2H); 2.26 (app t, 2H); 2.11 (m, 1H); 1.88 (m, 3H); 1.73 (s, 3H).

^{13}C NMR (125 MHz, $CDCl_3$): δ 141.8, 134.8, 128.6, 128.5, 126.0, 124.5, 47.1, 36.5, 34.4, 33.9, 30.6, 26.3, 16.2.

IR (NaCl plate, thin film, cm^{-1}): 3060, 3025, 2899, 2853, 1603, 1493, 1452, 1422, 1384, 1275, 1242, 1180, 1072, 1029, 908, 866, 742, 699.

HRMS-ESI (*m*/*z*): $[M + H]^+$ calculated for $C_{16}H_{22}S_2$, 279.1241; found, 279.1241.

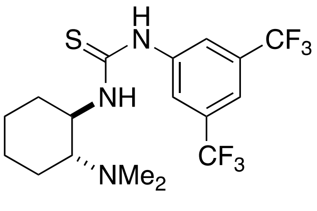

48

1-(3,5-Bis(trifluoromethyl)phenyl)-3-((1*R*,2*R*)-2-(dimethylamino)cyclohexyl)thiourea (48): As reported in the literature,[8] to a solution of 3,5-bis(trifluoromethyl)phenyl isothiocyanate (183 μL, 1.00 mmol, 1.0 equiv) in THF

(1 mL) was added (*R*,*R*)-*N*,*N*-dimethyl-*trans*-diaminocyclohexane[9,10] (142 mg, 1.00 mmol, 1.0 equiv). The reaction was stirred at rt overnight and then concentrated in vacuo. Silica gel chromatography (100:5:1 $CHCl_3$/MeOH/Et_3N) afforded the title compound as a white amorphous solid (300 mg, 72% yield), with spectroscopic data in agreement with literature values.

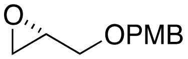

52

(*S*)-2-(((4-Methoxybenzyl)oxy)methyl)oxirane (52): To a suspension of NaH (60% dispersion in mineral oil, 2.40 g, 60.0 mmol, 2.0 equiv) in DMF (250 mL) at 0 °C was added (*R*)-glycidol (2.22 g, 30.0 mmol, 1.0 equiv). The reaction was stirred for 30 min at 0 °C, then 30 min at rt. Tetrabutylammonium iodide (5.54 g, 15.0 mmol, 0.50 equiv) was added, followed by PMBCl (8.54 mL, 63.0 mmol, 2.1 equiv). The reaction was stirred at rt overnight, then quenched with saturated aq. NH_4Cl (100 mL) and extracted with Et_2O (4 x 150 mL). The combined organics were dried over Na_2SO_4, filtered, and concentrated in vacuo. Silica gel chromatography (6:1 to 4:1 hexanes/EtOAc) afforded the title compound (4.55 g, 78%), with spectroscopic data in agreement with literature values.[11]

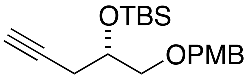

50

(*S*)-*tert*-Butyl((1-((4-methoxybenzyl)oxy)pent-4-yn-2-yl)oxy)dimethylsilane (50): In a glove box, a round bottom flask equipped with stir bar was charged with lithium acetylide–EDA (90%, 1.53 g, 15.0 mmol, 1.5 equiv). Outside the box under an Ar(g) atmosphere, THF (10 mL) was added, and the mixture cooled to 0 °C. DMSO (10 mL) was added, then epoxide **52** (1.94 g, 10.0 mmol, 1.0 equiv). The reaction was stirred at 0–5 °C for 4 h, then quenched with water, then saturated aq. NH_4Cl. The aqueous phase was extracted with Et_2O, and the organics were washed with saturated aq. $NaHCO_3$, then water. The combined organics were dried over $MgSO_4$, filtered, and

concentrated in vacuo. Silica gel chromatography (4:1 to 2:1 hexanes/EtOAc) afforded the alkynyl alcohol (1.85 g, 84% yield), which was carried on to the next step.

To a solution of alkynyl alcohol (1.85 g, 8.40 mmol, 1.0 equiv) in CH_2Cl_2 (42 mL) at 0 °C was added 2,6-lutidine (1.08 mL, 9.24 mmol, 1.1 equiv), followed by TBSOTf (2.12 mL, 9.24 mmol). The reaction was stirred for 2.5 h, gradually warming to rt. The reaction was quenched with saturated aq. NH_4Cl (50 mL) and extracted with EtOAc. The combined organics were dried over $MgSO_4$, filtered, and concentrated in vacuo. Silica gel chromatography (90:10 hexanes/EtOAc) afforded the title compound (2.77 g, 99% yield).

^{1}H NMR (500 MHz, $CDCl_3$): δ 7.26 (d, *J* = 8.7 Hz, 2H); 6.88 (d, *J* = 8.7 Hz, 2H); 4.48 (s, 2H); 3.97 (m, 1H); 3.82 (s, 3H); 3.49 (dd, *J* = 5.4, 9.7 Hz, 1H); 3.45 (dd, *J* = 5.4, 9. 7 Hz, 1H); 2.48 (ddd, *J* = 2.7, 5.9, 16.7 Hz, 1H); 2.36 (ddd, *J* = 2.7, 6.0, 16.7 Hz, 1H); 1.96 (t, *J* = 2.7 Hz, 1H); 0.89 (s, 9H); 0.10 (s, 3H); 0.08 (s, 3H).

^{13}C NMR (125 MHz, $CDCl_3$): δ 159.3, 130.6, 129.4, 113.9, 81.7, 73.5, 73.3, 70.5, 70.0, 55.5, 26.0, 24.9, 18.4, –4.4, –4.5.

IR (NaCl plate, thin film, cm^{-1}): 3311, 2954, 2930, 2857, 1613, 1514, 1464, 1362, 1302, 1249, 1173, 1124, 1038, 1007, 837, 778.

HRMS-ESI (*m*/*z*): $[M + Na]^+$ calculated for $C_{19}H_{30}O_3Si$, 357.1862; found, 357.1872.

$[\alpha]_D$ = –1.0 (*c* = 0.41, $CHCl_3$, 24 °C).

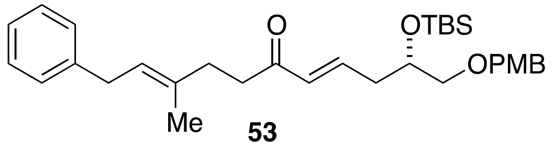

53

(*S*,2*E*,7*E*)-10-((*tert*-Butyldimethylsilyl)oxy)-11-((4-methoxybenzyl)oxy)-3-methyl-1-phenylundeca-2,7-dien-6-one (53): To a solution of alkyne **50** (1.22 g, 3.64 mmol, 1.0 equiv) in CH_2Cl_2 (30 mL) at 0 °C was added Schwartz's reagent, $Cp_2Zr(H)Cl$ (1.03 g, 4.01 mmol, 1.1 equiv) in several portions. The reaction was stirred for an additional 10 min after all of the solid had dissolved. The resulting yellow solution was cooled to –60 °C, and Me_2Zn (2.0 M in toluene, 2.09 mL, 4.19 mmol, 1.15 equiv) was added. The reaction mixture was stirred for 10 min at –60 °C, then moved to a cryocool bath at 0 °C. To this was added a solution of aldehyde **47** (823 mg, 4.37 mmol, 1.2 equiv) in CH_2Cl_2

(6 mL) via cannula over 15 min. The reaction was stirred at 0 °C overnight, then quenched with saturated aq. $NaHCO_3$ and extracted 4× with Et_2O. The combined organics were dried over $MgSO_4$, filtered, and concentrated in vacuo. Silica gel chromatography (80:20 hexanes/EtOAc) afforded the allylic alcohol (1.60 g, 84%).

To a solution of allylic alcohol (1.64 g, 3.12 mmol) in CH_2Cl_2 (31 mL) at 0 °C was added Dess–Martin periodinane (1.59 g, 3.75 mmol, 1.2 equiv). The reaction was stirred overnight, gradually warming to rt. The reaction was quenched with 1:1 saturated aq. $NaHCO_3/Na_2S_2O_3$ and extracted with EtOAc. The combined organics were dried over $MgSO_4$, filtered, and concentrated in vacuo. Silica gel chromatography (85:15 to 80:20 hexanes/EtOAc) afforded the title compound (1.24 g, 76%).

^{1}H NMR (500 MHz, $CDCl_3$): δ 7.29 (m, 2H); 7.24 (d, J = 8.7 Hz, 2H); 7.18 (m, 3H); 6.88 (d, J = 8.7 Hz, 2H); 6.84 (dt, J_d = 15.9 Hz, J_t = 7.5 Hz, 1H); 6.11 (d, J = 15.9 Hz, 1H); 5.36 (tq, J_t = 7.3 Hz, J_q = 1.3 Hz, 1H); 4.46 (d, J = 11.6 Hz, 1H); 4.43 (d, J = 11.6 Hz, 1H); 3.94 (m, 1H); 3.81 (s, 3H); 3.39 (dd, J = 5.3, 9.5 Hz, 1H); 3.36 (d, J = 7.3 Hz, 2H); 3.31 (dd, J = 6.3, 9.5 Hz, 1H); 2.65 (m, 2H); 2.49 (m, 1H); 2.36 (m, 3H); 1.74 (s, 3H); 0.87 (s, 9H); 0.04 (2 singlets, 6H).

^{13}C NMR (125 MHz, $CDCl_3$): δ 200.1, 159.4, 143.9, 135.2, 132.6, 130.4, 129.5, 128.6, 128.5, 126.0, 123.6, 113.9, 73.9, 73.2, 70.6, 55.4, 38.7, 38.2, 34.4, 34.0, 26.0, 18.3, 16.5, –4.3, –4.6.

IR (NaCl plate, thin film, cm^{-1}): 3028, 2929, 2857, 1697, 1673, 1613, 1514, 1463, 1362, 1302, 1250, 1173, 1101, 1036, 910, 836, 777, 734, 699.

HRMS-ESI (m/z): $[M + H]^+$ calculated for $C_{32}H_{46}O_4Si$, 523.3244; found, 523.3245.

$[\alpha]_D$ = –7.6 (c = 0.85, $CHCl_3$, 24 °C).

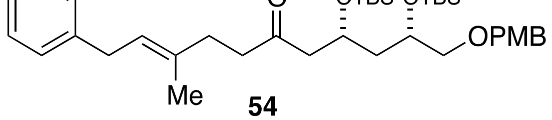

54

(8*R*,10*S*,*E*)-8,10-Bis((*tert*-butyldimethylsilyl)oxy)-11-((4-methoxybenzyl)oxy)-3-methyl-1-phenylundec-2-en-6-one (54): To MeOH (20 mL) at 0 °C was added HCl (200 μL). The mixture was stirred for 10 min, then a solution of enone **53** (1.05 g, 2.0 mmol, 1.0 equiv) in THF (5 mL) was added. The reaction was stirred at 0 °C

overnight, then quenched with saturated aq. $NaHCO_3$ (20 mL) and extracted with EtOAc (4 x 25 mL). The combined organics were dried over $MgSO_4$, filtered, and concentrated *in vacuo*. Silica gel chromatography (2:1 to 1:1 hexanes/EtOAc) afforded the alcohol as a colorless oil (560 mg, 68% yield), which was carried forward using one of two procedures.

Procedure A: To a solution of alcohol (102.1 mg, 0.25 mmol, 1.0 equiv), phenylboronic acid (38.1 mg, 0.31 mmol, 1.25 equiv), and powdered, dried 4 Å molecular sieves (250 mg) in toluene (2.5 mL) was added organocatalyst **48** (20.7 mg, 0.05 mmol, 20 mol %). The reaction was heated to 50 °C and stirred. After 48 h, the reaction was allowed to cool to rt, and was diluted with EtOAc (4 mL). A mixture of aq. H_2O_2 (150 μL) and saturated aq. K_2CO_3 (1.5 mL) was added, and the reaction was stirred for 15 min. To this was then added 5:1 saturated *aq*. $NaHCO_3/Na_2S_2O_3$ (6 mL). The reaction was extracted five times with EtOAc, then three times with CH_2Cl_2. The combined organics were dried over Na_2SO_4, filtered, and concentrated in vacuo. Silica gel chromatography (1:1 hexanes/EtOAc) afforded the *syn* diol (18.6 mg, 17%), along with recovered starting material (56.9 mg, 56%).

Procedure B: To a solution of alcohol (336 mg, 0.82 mmol, 1.0 equiv), phenylboronic acid (125 mg, 1.03 mmol, 1.25 equiv), and powdered, dried 4Å molecular sieves (850 mg) in toluene (8 mL) was added diisopropylamine (23 μL, 0.16 mmol, 20 mol %). The reaction was heated to 50 ºC and stirred. After 48 h, the reaction was allowed to cool to rt, and was diluted with EtOAc (10 mL). A mixture of aq. H_2O_2 (0.5 mL) and saturated aq. Na_2CO_3 (5 mL) was added, and the reaction was stirred for 15 min. To this was then added 5:1 saturated aq. $NaHCO_3/Na_2S_2O_3$ (12 mL). The reaction was extracted five times with EtOAc, then twice with CH_2Cl_2. The combined organics were dried over Na_2SO_4, filtered, and concentrated in vacuo. Silica gel chromatography (1:1 to 1:2 hexanes/EtOAc) afforded the *syn* diol (80.1 mg, 23%), along with the *anti* diastereomer (62.3 mg, 17%).

To a solution of the *syn* diol (54.5 mg, 0.128 mmol, 1.0 equiv) in CH_2Cl_2 (2.4 mL) at 0 °C was added 2,6-lutidine (36 μL, 0.31 mmol, 2.4 equiv), then TBSOTf (65 μL, 0.28 mmol, 2.2 equiv). The reaction was stirred overnight, gradually warming to rt. The reaction was quenched with saturated aq. $NaHCO_3$, then extracted with CH_2Cl_2 (4 ×

20 mL). The combined organics were dried over $MgSO_4$, filtered, and concentrated in vacuo*.* Silica gel chromatography (95:5 to 90:10 hexanes/EtOAc) afforded the title compound (41.7 mg, 50%).

^{1}H NMR (500 MHz, $CDCl_3$): δ 7.29 (m, 2H); 7.26 (d, *J* = 8.7 Hz, 2H); 7.17 (m, 3H); 6.88 (d, *J* = 8.7 Hz, 2H); 5.35 (tq*,* J_t = 7.3 Hz, J_q = 1.3 Hz, 1H); 4.45 (s, 2H); 4.33 (m, 1H); 3.86 (m, 1H); 3.81 (s, 3H); 3.36 (m, 4H); 2.57 (m, 4H); 2.29 (app t, 2H); 1.72 (s, 3H); 1.68 (m, 2H); 0.89 (s, 9H); 0.85 (s, 9H); 0.07 (s, 3H); 0.05 (2 singlets, 6H); 0.02 (s, 3H).

^{13}C NMR (125 MHz, $CDCl_3$): δ 209.5, 159.3, 141.6, 135.2, 130.7, 129.4, 128.6, 128.5, 126.0, 123.6, 113.9, 79.8, 73.1, 69.0, 66.8, 55.5, 50.1, 43.6, 43.1, 34.4, 33.2, 26.1, 26.0, 18.3, 18.1, 16.5, –4.3, –4.5, –4.6 (2 peaks).

IR (NaCl plate, thin film, cm^{-1}): 3028, 2954, 2929, 2856, 1716, 1613, 1587, 1514, 1495, 1472, 1361, 1303, 1250, 1173, 1100, 1039, 1006, 910, 837, 777, 736, 698.

HRMS-ESI (*m*/*z*): $[M + H]^+$ calculated for $C_{38}H_{62}O_5Si_2$, 677.4034; found, 677.4023.

$[\alpha]_D$ = –13.7 (*c* = 2.4, $CHCl_3$, 23 ° C).

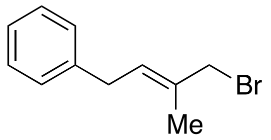

56

(*E*)-(4-Bromo-3-methylbut-2-en-1-yl)benzene (56): To a solution of triphenylphosphine (1.97 g, 7.5 mmol, 1.5 equiv) in CH_2Cl_2 (50 mL) at 0 °C was added carbon tetrabromide (2.49 g, 7.5 mmol, 1.5 equiv). To this, (*E*)-2-methyl-4-phenyl-but-2-en-1-ol[12] (811 mg, 5.0 mmol, 1.0 equiv) in CH_2Cl_2 (50 mL) was added dropwise. The reaction was stirred for 1 h at 0 °C, then quenched with saturated aq. $NaHCO_3$. The organic phase was separated, and the aqueous phase extracted twice with hexanes. The combined organics were dried over $MgSO_4$, filtered, and concentrated in vacuo. The residue was triturated with hexanes and filtered. The filtrate was concentrated in vacuo*.* Silica gel chromatography (hexanes to 95:5 hexanes/EtOAc) afforded the title compound (515 mg, 46%, 17:1 *E*:*Z*).

^{1}H NMR (500 MHz, $CDCl_3$): 7.31 (m, 2H); 7.22 (m, 1H); 7.18 (m, 2H); 5.81 (tq, J_t = 7.4 Hz, J_q = 0.7 Hz, 1H); 4.02 (s, 2H); 3.41 (d, *J* = 7.4 Hz, 2H); 1.90 (s, 3H).

^{13}C NMR (125 MHz, $CDCl_3$): 140.3, 133.1, 129.9, 128.8, 128.5, 126.4, 41.5, 34.7, 15.1.
IR (NaCl plate, thin film, cm^{-1}): 3084, 3062, 3027, 2918, 1659, 1603, 1494, 1453, 1434, 1387, 1205, 1144, 1075, 1029, 1003, 904, 783, 742, 671.

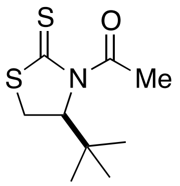

58

(*S*)-1-(4-*tert*-Butyl)-2-thioxothiazolidin-3-yl)ethanone (58): In a 1L round bottom flask equipped with reflux condenser, dry ice condenser, Ar(g) inlet, and outlet to an aqueous bleach trap, L-*tert*-leucinol (10.0 g, 85.3 mmol, 1.0 equiv) was dissolved in aq. KOH (5M, 400 mL). To this was added CS_2 (41.1 mL, 683 mmol, 8.0 equiv). The reaction was heated to 110 °C (bath temperature) for 12 h and allowed to cool to rt overnight. The addition of CS_2 (41.1 mL, 683 mmol, 8.0 equiv), heating, and cooling was repeated two more times. The reaction was then extracted with CH_2Cl_2 (3 x 400 mL). The combined organics were dried over $MgSO_4$, filtered, and concentrated in vacuo. Silica gel chromatography (90:10 CH_2Cl_2/MeOH) afforded (4*S*)-*tert*-butyl-thiazolidine-2-thione as a white solid (7.44 g, 50%).

To a solution of (4*S*)-*tert*-butyl-thiazolidine-2-thione (7.08 g, 40.4 mmol, 1.0 equiv) in THF (200 mL) at –78 °C was added *n*-BuLi (2.5 M in hexanes, 17.8 mL, 44.4 mmol, 1.1 equiv) dropwise. The mixture was stirred for 30 min at –78 °C, and then acetyl chloride (3.16 mL, 44.4 mmol, 1.1 equiv) was added. The reaction was stirred for 4 h at –78 °C, then warmed to rt and stirred for an additional 30 min. The reaction was then cooled to 0 °C and quenched with saturated aq. NH_4Cl. The organic phase was separated, and the aqueous phase was further extracted twice with CH_2Cl_2. The combined organics were dried over $MgSO_4$, filtered, and concentrated in vacuo. Silica gel chromatography (1:1 hexanes/CH_2Cl_2) afforded the title compound as a yellow oil (7.63 g, 87%), with spectroscopic data in agreement with literature values.[13]

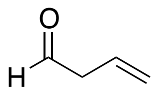

59

But-3-enal (59): Octa-1,7-diene-4,5-diol[14] (853 mg, 6.0 mmol, 1.0 equiv) was dissolved in a 1:1 mixture of CH_2Cl_2 and aqueous pH = 4 phosphate buffer (12 mL). The mixture was cooled to 0 °C, and $NaIO_4$ was added (1.28 g, 6.0 mmol, 1.0 equiv). The reaction was transferred to a stir plate in a 4 °C refrigerator and stirred vigorously overnight. After 16 h, the organic phase was separated and then washed with sat. aq. Na_2SO_3 solution and brine. The aqueous phase was extracted once with a minimum volume of CH_2Cl_2. The organics were dried over $MgSO_4$ and filtered. A ^{1}H NMR spectrum was taken of the CH_2Cl_2 solution to confirm that diol cleavage proceeded to >95% conversion. The solution was used immediately in the subsequent aldol reaction.

^{1}H NMR (500 MHz, $CDCl_3$): δ 9.71 (t, J = 1.9 Hz, 1H); 5.93 (ddt, J_t = 6.9 Hz, J_d = 10.3, 17.2 Hz, 1H); 5.30 (partially obscured by CH_2Cl_2, 1H); 5.23 (dq, J_q = 1.5 Hz, J_d = 17.2 Hz, 1H); 3.21 (dm, J_d = 6.9 Hz, 2H).

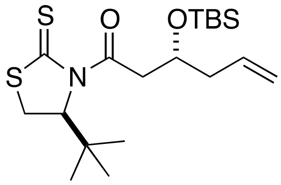

60

(*R*)-1-((*S*)-4-(*tert*-Butyl)-2-thioxothiazolidin-3-yl)-3-((*tert*-butyldimethylsily)oxy)hex-5-en-1-one (60): To a solution of thiazolidinethione **58** (908 mg, 4.18 mmol, 1.0 equiv) in CH_2Cl_2 (21 mL) at rt was added dichlorophenylborane (650 μL, 5.01 mmol, 1.2 equiv). The orange solution was stirred for 10 min, then (–)-sparteine (2.30 mL, 10.0 mmol, 2.4 equiv) was added. The reaction was stirred for 30 min at rt, then cooled to −78 °C. A cold solution of but-3-enal (**59**) in CH_2Cl_2 (*ca*. 6.0 mmol, 1.4 equiv) was added via cannula. The reaction was stirred for 4 h at –78 °C, warmed to rt over 1 h, and stirred 30 min at rt. The reaction was diluted with hexanes (30 mL), then quenched with *aq*. H_2O_2 (30%, 20 mL). The reaction was diluted with 4:1 hexanes/CH_2Cl_2 (400 mL), and the organics were washed with water, followed by brine. The organics

were dried over $MgSO_4$, filtered, and concentrated in vacuo. Silica gel chromatography (5:1 CH_2Cl_2/hexanes to CH_2Cl_2) afforded the aldol adduct as a yellow oil (604 mg, 50%).

To a solution of aldol adduct (287 mg, 1.0 mmol, 1.0 equiv) in CH_2Cl_2 (10 mL) at 0 °C was added 2,6-lutidine (128 μL, 1.1 mmol, 1.1 equiv), followed by TBSOTf (253 μL, 1.1 mmol, 1.1 equiv). The reaction was stirred for 5 h, gradually warming to rt. The reaction was quenched with brine and extracted three times with CH_2Cl_2. The combined organics were dried over $MgSO_4$, filtered, and concentrated in vacuo. Silica gel chromatography (90:10 hexanes/EtOAc) afforded the title compound as a yellow oil (316 mg, 79%).

^{1}H NMR (500 MHz, $CDCl_3$): δ 5.83 (m, 1H); 5.31 (dd, *J* = 0.7, 8.3 Hz, 1H); 5.07 (app dd); 4.32 (pentet, *J* = 5.7 Hz, 1H); 3.54 (dd, *J* = 5.5, 17.4 Hz, 1H); 3.50 (dd, *J* = 8.3, 11.8 Hz, 1H); 3.34 (dd, *J* = 6.4, 17.4 Hz, 1H); 3.10 (dd, *J* = 0.7, 11.8 Hz, 1H); 2.28 (m, 2H); 1.04 (s, 9H); 0.88 (s, 9H); 0.09 (s, 3H); 0.07 (s, 3H).

^{13}C NMR (125 MHz, $CDCl_3$): δ 205.0, 171.1, 134.7, 117.8, 72.4, 68.5, 45.4, 42.4, 38.1, 30.6, 27.1, 26.1, 18.2, –4.3, –4.4.

IR (NaCl plate, thin film, cm^{-1}): 2958, 2930, 2857, 1700, 1472, 1370, 1318, 1254, 1158, 1136, 1090, 911, 837, 777, 735.

HRMS-ESI (*m*/*z*): $[M + Na]^+$ calculated for $C_{19}H_{35}NO_2S_2Si$, 424.1771; found 424.1782.

$[\alpha]_D$ = +229.0 (*c* = 0.925, $CHCl_3$, 24 °C).

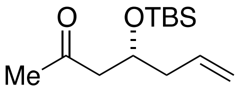

57

(*R*)-4-((*tert*-Butyldimethylsilyl)oxy)hept-6-en-2-one (57): In a 5 mL Biotage microwave vial equipped with stir bar, a solution of thiazolidinethione **60** (214.8 mg, 0.53 mmol, 1.0 equiv) was dissolved in CH_2Cl_2 (3.5 mL). To this was added imidazole (54.6 mg, 0.80 mmol, 1.5 equiv), followed by *N*,*O*-dimethylhydroxylamine hydrochloride (62.6 mg, 0.64 mmol, 1.2 equiv). The vial was sealed and irradiated to 100 °C with stirring for 15 min using a Biotage Initiator Eight microwave reactor. The vial was opened, and the reaction was diluted with water and extracted with CH_2Cl_2. Silica gel

chromatography (90:10 to 80:20 hexanes/EtOAc) afforded the Weinreb amide (91.2 mg, 59%).

To a solution of Weinreb amide (86.2 mg, 0.30 mmol) in Et_2O (3.0 mL) at 0 °C was added methylmagnesium chloride (3.0 M in THF, 0.30 mL, 9.0 mmol, 3.0 equiv) dropwise. The reaction was stirred for 1h at 0 °C, then quenched with saturated *aq*. NH_4Cl and extracted with Et_2O. The combined organics were dried over $MgSO_4$, filtered, and concentrated in vacuo. Silica gel chromatography (90:10 hexanes/EtOAc) afforded the title compound (56.2 mg, 77%) as a colorless oil.

^{1}H NMR (500 MHz, $CDCl_3$): 5.80 (dddd, *J* = 7.1, 7.3, 10.7, 16.7 Hz, 1H); 5.06 (m, 2H); 4.24 (m, 1H); 2.60 (dd, *J* = 7.3, 15.5 Hz, 1H); 2.49 (dd, *J* = 5.0, 15.5 Hz, 1H); 2.25 (m, 2H); 2.16 (s, 3H); 0.87 (s, 9H); 0.08 (s, 3H); 0.04 (s, 3H).

^{13}C NMR (125 MHz, $CDCl_3$): 208.2, 134.5, 117.9, 68.7, 50.5, 42.4, 32.0, 26.0, –4.3, –4.6.

IR (NaCl plate, thin film, cm^{-1}): 2957, 2930, 2857, 1718, 1473, 1361, 1167, 1093, 1006, 915, 837, 777.

HRMS-ESI (*m*/*z*): $[M + Na]^+$ calculated for $C_{13}H_{26}O_2Si$, 265.1600; found, 265.1602.

$[\alpha]_D$ = –36.8 (*c* = 0.19, $CHCl_3$, 24 °C).

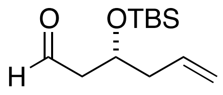

62

(*R*)-3-((*tert*-Butyldimethylsilyl)oxy)hex-5-enal (62): To a solution of thiazolidinethione **60** (184.6 mg, 0.46 mmol, 1.0 equiv) in CH_2Cl_2 (5.0 mL) at –78 °C, DIBAL-H (1.0 M in CH_2Cl_2, 0.51 mL, 0.51 mmol, 1.1 equiv) was added dropwise. The reaction was stirred for 40 min at –78 °C. A second, equivalent portion of DIBAL-H was added and the reaction stirred for an additional 10 min at –78 °C. The reaction was quenched with the addition of MeOH, followed by saturated aq. Rochelle's salt solution. The organic phase was separated, and the aqueous phase extracted with CH_2Cl_2. The combined organics were dried over $MgSO_4$, filtered, and concentrated in vacuo. Silica gel chromatography (95:5 hexanes/EtOAc) afforded the title compound (67.1 mg, 64%).

^{1}H NMR (500 MHz, $CDCl_3$): 9.81 (t, J = 2.4 Hz, 1H); 5.79 (dddd, J = 7.2, 7.2, 10.4, 17.6, 1H); 5.09 (m, 2H); 4.27 (pentet, J = 5.9 Hz, 1H); 2.54 (m, 2H); 2.32 (m, 2H); 0.88 (s, 9H); 0.09 (s, 3H); 0.07 (s, 3H).

^{13}C NMR (125 MHz, $CDCl_3$): 202.4, 134.0, 118.4, 67.9, 50.6, 42.5, 26.0, 18.2, –4.2, –4.6.

IR (NaCl plate, thin film, cm^{-1}): 2957, 2930, 2897, 2858, 1728, 1473, 1464, 1362, 1257, 1101, 1038, 1005, 917, 837, 777.

HRMS-ESI (m/z): $[M + Na]^+$ calculated for $C_{12}H_{24}O_2Si$, 251.1438; found, 251.1444.

$[\alpha]_D$ = –10.2 (c = 0.42, $CHCl_3$, 24 °C).

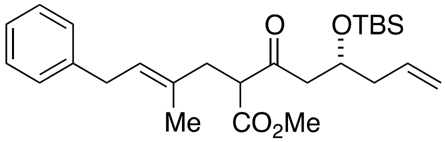

61

(5*R*)-Methyl 5-((*tert*-butyldimethylsilyl)oxy)-2-((*E*)-2-methyl-4-phenylbut-2-en-1-yl)-3-oxooct-7-enoate (61): To a solution of methyl ester **45** (240.1 mg, 1.1 mmol, 1.1 equiv) in THF (11 mL) at –78 °C was added LDA (1.8 M in heptanes/THF, 0.61 mL, 1.1 mmol, 1.1 equiv). The mixture was stirred 30 min, and a solution of aldehyde **62** (229.0 mg, 1.0 mmol, 1.0 equiv) in THF (2 mL) was added. The reaction was stirred for 2 h at −78 °C, then was removed from its cooling bath and immediately quenched with saturated aq. NH_4Cl. Water was added to dissolve the resulting salts, and the aqueous phase was extracted with Et_2O. The combined organics were dried over $MgSO_4$, filtered, and concentrated in vacuo. Silica gel chromatography (95:5 to 90:10 to 80:20 hexanes/EtOAc) afforded the aldol adduct (385.1 mg, 86%) as a mixture of diastereomers, which was carried forward to the next step.

To a solution of aldol adduct (385.1 mg, 0.86 mmol, 1.0 equiv) in CH_2Cl_2 (4.3 mL) was added 4 Å molecular sieves (43 mg), followed by NMO (202.0 mg, 1.72 mmol, 2.0 equiv), then TPAP (30.3 mg, 0.086 mmol, 10 mol %). The reaction was stirred for 2 h at rt, then filtered through a pad of silica, washing with CH_2Cl_2. The filtrate was concentrated in vacuo. Silica gel chromatography (95:5 to 90:10 hexanes/EtOAc)

afforded the title compound as an undefined mixture of diastereomers at the central carbon of the β-ketoester (257.9 mg, 67%).

^{1}H NMR (500 MHz, $CDCl_3$): 7.27 (m, 2H); 7.18 (m, 1H); 7.13 (m, 2H); 5.77 (m, 1H); 5.39 (t, J = 7.4 Hz, 1H); 5.05 (m, 2H); 4.28-4.13 (two multiplets, 1H); 3.68 (m, 1H); 3.63 (two singlets, 3H); 3.33 (m, 2H); 2.69 (app ddd, 1H); 2.56 (m, 3H); 2.23 (m, 2H); 1.73 (s, 3H); 0.86 (s, 9H); 0.07 (app d, 3H); 0.03-0.02 (two singlets, 3H).

^{13}C NMR (125 MHz, $CDCl_3$): 203.3, 203.1, 170.0, 168.0, 141.2, 134.5, 134.2, 132.3, 128.6, 128.4 (2 peaks), 126.1 (2 peaks), 126.0, 122.6, 118.0, 117.9, 68.1, 67.7, 59.1, 58.5, 52.4 (2 peaks), 49.0, 48.5, 42.1, 42.0, 37.8, 37.6, 34.4, 26.0 (2 peaks), 18.2 (2 peaks), 16.3, 16.2, –4.4 (2 peaks), –4.6 (2 peaks).

IR (NaCl plate, thin film, cm^{-1}): 2954, 2930, 2857, 1746, 1718, 1642, 1495, 1436, 1361, 1255, 1162, 1077, 1004, 915, 837, 811, 777, 735, 698.

$[\alpha]_D$ = –23.1 (c = 0.595, $CHCl_3$, 24 °C).

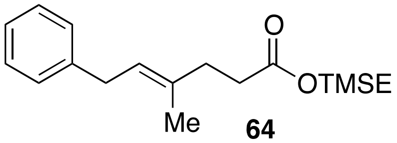

64

(*E*)-2-(Trimethylsilyl)ethyl 4-methyl-6-phenylhex-4-enoate (64): Methyl ester **45** (3.37 g, 15.4 mmol, 1.0 equiv) and 2-(trimethylsilyl)ethanol (17.7 mL, 123 mmol, 8.0 equiv) were dissolved in DME (20 mL) in a 100 mL round bottom flask equipped with stir bar and reflux condenser. To this was added $Ti(OiPr)_4$ (1.14 mL, 3.85 mmol, 0.25 equiv) in one portion. The reaction was heated to 120 °C in an oil bath for 48 hours. After this time, the mixture was allowed to cool to rt, then was quenched with saturated aq. NH_4Cl and extracted with pentane. The combined organics were dried over $MgSO_4$, filtered, and concentrated in vacuo. Silica gel chromatography (95:5 hexanes/EtOAc) afforded the title compound as a colorless liquid (3.79 g, 81%).

^{1}H NMR (500 MHz, $CDCl_3$): δ 7.28 (m, 2H); 7.18 (m, 3H); 5.39 (tq, J_t = 7.3 Hz, J_q = 1.3 Hz, 1H); 4.14 (m, 2H); 3.36 (d, J = 7.3 Hz, 2H); 2.44 (m, 2H); 2.37 (m, 2H); 1.74 (s, 3H); 0.96 (m, 2H); 0.04 (s, 9H).

^{13}C NMR (125 MHz, $CDCl_3$): δ 173.7, 141.6, 134.8, 128.6, 128.5, 126.0, 124.0, 62.7, 34.9, 34.3, 33.5, 17.5, 16.4, 1.3.

IR (NaCl plate, thin film, cm^{-1}): 3028, 2954, 1734, 1604, 1494, 1453, 1383, 1344, 1250, 1161, 1062, 939, 860, 838, 742, 698.
HRMS-ESI (*m*/*z*): $[M + Na]^+$ calculated for $C_{18}H_{28}O_2Si$, 327.1751; found, 327.1744.

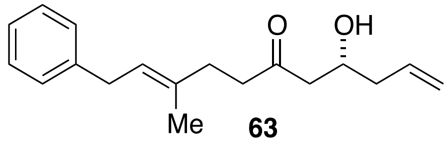

63

(*R*,*E*)-4-Hydroxy-9-methyl-11-phenylundeca-1,9-dien-6-one (63): To a solution of TMSE ester **64** (1.61 g, 5.30 mmol, 1.1 equiv) in THF (53 mL) at –78 °C was added LDA (1.8 M in heptanes/THF, 2.94 mL, 5.30 mmol, 1.1 equiv). The mixture was stirred 30 min, and a solution of aldehyde **62** (1.10 g, 4.82 mmol, 1.0 equiv) in THF (10 mL) was added. The reaction was stirred for 2 h at –78 °C, then was removed from its cooling bath and immediately quenched with saturated aq. NH_4Cl. Water was added to dissolve the resulting salts, and the aqueous phase was extracted with Et_2O. The combined organics were dried over $MgSO_4$, filtered, and concentrated in vacuo. Silica gel chromatography (95:5 to 90:10 hexanes/EtOAc) afforded the aldol adduct (1.874 g, 86%) as a mixture of diastereomers, which was carried forward to the next step.

To a solution of aldol adduct (1.874 g, 3.62 mmol, 1.0 equiv) in CH_2Cl_2 (11 mL) was added 4Å molecular sieves (1.81 g), followed by NMO (848 mg, 7.24 mmol, 2.0 equiv), then TPAP (63.6 mg, 0.18 mmol, 5 mol %). The reaction was stirred for 2 h at rt, then filtered through a pad of silica, washing with CH_2Cl_2, then EtOAc. The filtrate was concentrated in vacuo. Silica gel chromatography (95:5 to 90:10 hexanes/EtOAc) afforded the β-ketoester (1.214 g, 65%).

To a solution of β-ketoester (734.8 mg, 1.42 mmol, 1.0 equiv) in THF (14 mL) at 0 °C was added TBAF (1.0 M in THF, 3.56 mL, 3.56 mmol, 2.5 equiv). The reaction was stirred overnight, warming to rt, then was quenched with brine. The aqueous phase was extracted twice with Et_2O, then twice with EtOAc. The combined organics were dried over Na_2SO_4, filtered, and concentrated *in vacuo*. Silica gel chromatography (90:10 to 80:20 hexanes/EtOAc) afforded the title compound (197.9 mg, 51%).

^1H NMR (500 MHz, $CDCl_3$): 7.29 (m, 2H); 7.18 (m, 3H); 5.81 (m, 1H); 5.34 (t, *J* = 7.3 Hz, 1H); 5.12 (m, 2H); 4.10 (m, 1H); 3.35 (d, *J* = 7.4 Hz, 2H); 2.99 (d, *J* = 3.1 Hz,

1H); 2.62 (dd, J = 3.0, 17.5 Hz, 1H); 2.58 (app t, 2H); 2.53 (dd, J = 9.0, 17.5 Hz, 1H); 2.33 (app t, 2H); 2.24 (m, 2H); 1.73 (s, 3H).
^{13}C NMR (125 MHz, $CDCl_3$): 211.7, 141.5, 134.4, 128.6, 128.5, 126.0, 124.1, 118.2, 67.2, 48.5, 42.3, 41.1, 34.4, 33.4, 16.5.
IR (NaCl plate, thin film, cm^{-1}): 3447, 3063, 3027, 2977, 2917, 1709, 1641, 1603, 1494, 1453, 1435, 1408, 1384, 1049, 998, 917, 742, 699.
HRMS-ESI (*m*/*z*): $[M + Na]^+$ calculated for $C_{18}H_{24}O_2$, 295.1674; found, 295.1668.
$[\alpha]_D$ = –20.9 (c = 0.41, $CHCl_3$, 24 °C).

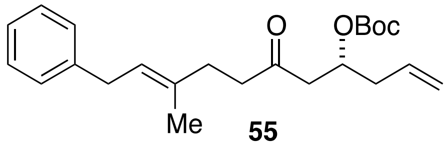

55

(*R*,*E*)-*tert*-Butyl (9-methyl-6-oxo-11-phenylundeca-1,9-dien-4-yl) carbonate (55): To a solution of compound **63** (40.1 mg, 0.147 mmol, 1.0 equiv) in CH_2Cl_2 at 0 °C were added pyridine (59 μL, 0.74 mmol, 5.0 equiv), DMAP (4.5 mg, 0.037 mmol, 25 mol %), and di-*tert*-butyl dicarbonate (68 μL, 0.29 mmol, 2.0 equiv). The reaction was stirred overnight, gradually warming to rt, then quenched with saturated aq. $NaHCO_3$ and extracted three times with CH_2Cl_2. The combined organics were washed with saturated aq. NH_4Cl, then dried over $MgSO_4$, filtered, and concentrated in vacuo. Silica gel chromatography (90:10 to 80:20 hexanes/EtOAc) afforded the title compound as a colorless oil (25.5 mg, 47%).
^{1}H NMR (500 MHz, $CDCl_3$): 7.29 (m, 2H); 7.17 (m, 3H); 5.76 (m, 1H); 5.34 (tq, J_t = 7.3 Hz, J_q = 1.3 Hz, 1H); 5.14 (m, 1H); 5.10 (m, 2H); 3.35 (d, J = 7.3 Hz, 2H); 2.77 (dd, J = 7.4, 16.8 Hz, 1H); 2.62 (dd, J = 5.4, 16.7 Hz, 1H); 2.55 (m, 2H); 2.38 (app t, 2H); 2.30 (t, J = 7.7 Hz, 2H); 1.72 (s, 3H); 1.47 (s, 9H).
^{13}C NMR (125 MHz, $CDCl_3$): 207.2, 153.0, 141.6, 134.8, 133.1, 128.6, 128.5, 126.0, 123.9, 118.8, 82.4, 72.3, 46.3, 42.1, 38.7, 34.4, 33.3, 28.0, 16.5.
IR (NaCl plate, thin film, cm^{-1}): 3082, 3063, 3027, 2980, 2933, 1739, 1643, 1604, 1495, 1453, 1369, 1279, 1163, 1092, 1039, 994, 917, 839, 792, 735, 699.
HRMS-ESI (*m*/*z*): $[M + Na]^+$ calculated for $C_{23}H_{32}O_4$, 395.2198; found, 395.2206.
$[\alpha]_D$ = –5.5 (c = 0.835, $CHCl_3$, 24 °C).

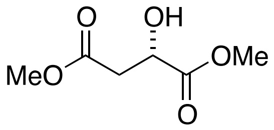

68

(*S*)-Dimethyl 2-hydroxysuccinate (68): To a 2 L flask charged with stir bar and anhydrous methanol (500 mL) was added acetyl chloride (22.4 mL, 315 mmol, 0.6 equiv) dropwise. The resultant solution was stirred for 10 min at rt, then (*S*)-malic acid (70.55 g, 526 mmol) was added. The reaction was stirred overnight at rt, then concentrated in vacuo. Silica gel chromatography (96:4 CH_2Cl_2/MeOH) afforded the title compound as a pale yellow oil (80.57 g, 94%), with spectroscopic data in agreement with literature values.[15]

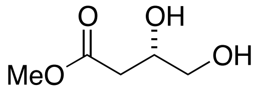

69

(*S*)-Methyl-3,4-dihydroxybutanoate (69): As reported in the literature,[16] to a solution of diester **68** (31.72 g, 195.6 mmol, 1.00 equiv) in THF (375 mL) was added borane-dimethyl sulfide (19.0 mL, 200 mmol, 1.02 equiv). The reaction was stirred at room temperature for 30 min. Sodium borohydride (378 mg, 10.0 mmol, 0.05 equiv) was added, and the reaction was stirred for an additional 30 min, followed by the addition of MeOH (anhydrous, 125 mL). Following 30 min of continued stirring, the reaction mixture was concentrated in vacuo. The concentrate was purified by silica gel chromatography (EtOAc) to afford the title compound as a colorless oil (20.19 g, 77% yield), with spectroscopic data in agreement with literature values.[15]

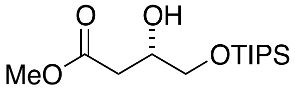

S1

(*S*)-Methyl 3-hydroxy-4-((triisopropylsilyl)oxy)butanoate (S1): As reported in the literature,[17,18] diol **69** (9.85 g, 73.4 mmol, 1.0 equiv) was dissolved in CH_2Cl_2 (350 mL) and cooled to 0 °C. To this was added 2,6-lutidine (9.4 mL, 80.8 mmol, 1.1 equiv),

followed by TIPS triflate (20.7 mL, 77.1 mmol, 1.05 equiv). The reaction was stirred 1 h at 0 °C. The reaction was quenched with saturated aq. NH_4Cl, and the aqueous layer was extracted with CH_2Cl_2. The combined organics were dried over $MgSO_4$, filtered, and concentrated in vacuo. Silica gel chromatography (85:15 hexanes/EtOAc) afforded the title compound (16.64 g, 78%).

^{1}H NMR (500 MHz, $CDCl_3$): δ 4.12 (m, 1H); 3.73 (dd, *J* = 4.9, 9.8 Hz, 1H); 3.72 (s, 3H); 3.67 (dd, *J* = 5.9, 9.8 Hz, 1H); 2.9 (d, *J* = 4.8 Hz, 1H); 2.58 (dd, *J* = 5.0, 16.0 Hz, 1H); 2.53 (dd, *J* = 7.7, 16.0 Hz, 1H); 1.07 (m, 21H).

^{13}C NMR (125 MHz, $CDCl_3$): δ 172.7, 68.9, 66.6, 52.0, 38.1, 18.1, 12.1.

IR (NaCl plate, thin film, cm^{-1}): 3482, 2945, 2893, 2867, 1741, 1464, 1438, 1257, 1170, 1123, 1065, 883, 797, 683.

HRMS-ESI (*m*/*z*): $[M + H]^+$ calculated for $C_{14}H_{30}O_4Si$, 291.1986; found, 291.1988.

$[\alpha]_D$ = –8.6 (*c* = 1.5, $CHCl_3$, 24 °C).

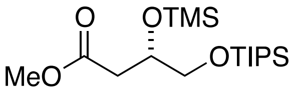

70

(*S*)-Methyl 4-((triisopropylsilyl)oxy)-3-((trimethylsilyl)oxy)butanoate (70): Hydroxy ester **S1** (17.04 g, 58.7 mmol, 1.0 equiv) was dissolved in CH_2Cl_2 (400 mL) and cooled to 0 °C. Triethylamine (18.0 mL, 129 mmol, 1.1 equiv) was added, followed by TMSCl (8.2 mL, 64.5 mmol, 2.2 equiv). The reaction was stirred for 3 h, gradually warming to room temperature. The reaction was quenched with sat. aq. NH_4Cl, and the aqueous layer was extracted with CH_2Cl_2. The combined organics were dried over Na_2SO_4, filtered, and concentrated in vacuo. Silica gel chromatography (95:5 hexanes/EtOAc) afforded the title compound as a colorless oil (20.18 g, 95%).

^{1}H NMR (500 MHz, $CDCl_3$): δ 4.18 (dddd, *J* = 3.9, 5.3, 7.3, 8.6 Hz, 1H); 3.69 (s, 3H); 3.67 (dd, *J* = 5.3, 9.7 Hz, 1H); 3.49 (dd, *J* = 7.3, 9.7 Hz, 1H); 2.72 (dd, *J* = 3.9, 15.0 Hz, 1H); 2.39 (dd, *J* = 8.6, 15.0 Hz, 1H); 1.06 (m, 21H); 0.11 (s, 9H).

^{13}C NMR (125 MHz, $CDCl_3$): δ 172.6, 70.6, 67.4, 51.7, 40.3, 18.2, 12.1, 0.4.

IR (NaCl plate, thin film, cm^{-1}): 2946, 2894, 2867, 1744, 1463, 1438, 1251, 1171, 1124, 1088, 1013, 997, 883, 843, 804, 683.

HRMS-ESI (*m*/*z*): $[M + H]^+$ calculated for $C_{17}H_{38}O_4Si_2$, 363.2381; found, 363.2385.
$[\alpha]_D$ = –26.3 (*c* = 1.2, $CHCl_3$, 23 °C).

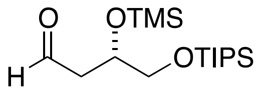

71

(*S*)-4-((Triisopropylsilyl)oxy)-3-((trimethylsilyl)oxy)butanal (71): To a solution of ester **70** (10.39 g, 28.7 mmol, 1.0 equiv) in Et_2O (250 mL) at –78 °C was added DIBAL-H (1.0 M in CH_2Cl_2, 57.3 mL, 57.3 mmol, 2.0 equiv) dropwise via addition funnel. (Note: Use of 1.0 equiv of DIBAL-H led to an inseparable mixture of aldehyde and recovered ester.) The reaction was stirred at –78 °C for 2 h, then quenched with MeOH (5 mL) and sat. aq. Rochelle's salt solution. The mixture was allowed to warm to room temperature and stir until phase separation was observed. The aqueous layer was extracted three times with Et_2O. The combined organics were dried over $MgSO_4$, filtered, and concentrated in vacuo. Silica gel chromatography (95:5 hexanes/EtOAc) afforded the title compound as a colorless oil (7.72 g, 81%).

^{1}H NMR (500 MHz, $CDCl_3$): δ 9.82 (t, *J* = 2.3 Hz, 1H); 4.24 (dddd, *J* = 4.9, 4.9, 7.3, 7.3 Hz, 1H); 3.73 (dd, *J* = 5.0, 9.7 Hz, 1H); 3.53 (dd, *J* = 7.4, 9.7 Hz, 1H); 2.72 (ddd, *J* = 2.1, 4.7, 15.9 Hz, 1H); 2.55 (ddd, *J* = 2.7, 7.2, 15.9 Hz, 1H); 1.06 (m, 21H); 0.13 (s, 9H).
^{13}C NMR (125 MHz, $CDCl_3$): δ 201.9, 69.0, 67.5, 49.1, 18.2, 12.1, 0.4.
IR (NaCl plate, thin film, cm^{-1}): 2946, 2868, 2724, 1731, 1464, 1384, 1252, 1100, 1070, 1013, 882, 843, 682.
HRMS-ESI (*m*/*z*): $[M + H]^+$ calculated for $C_{16}H_{36}O_3Si_2$, 333.2276; found, 333.2279.
$[\alpha]_D$ = –19.3 (*c* = 1.0, $CHCl_3$, 24 °C).

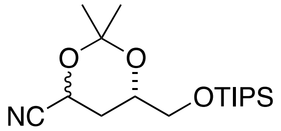

67

(6*S*)-2,2-Dimethyl-6-(((triisopropylsilyl)oxy)methyl)-1,3-dioxane-4-carbonitrile (67): To aldehyde **71** (10.87 g, 32.7 mmol, 1.0 equiv) at 0 °C were added TMSCN (4.25 mL, 32.7 mmol, 1.0 equiv), KCN (1 mg), and 18-crown-6 (4 mg). The reaction was stirred for

1 h, warming to rt. Acetone (80 mL) and 2,2-dimethoxypropane (20 mL) were added, followed by camphorsulfonic acid (125 mg). The reaction mixture was stirred 48 h at rt. Triethylamine (5 mL) was added and the mixture stirred for 10 min, then concentrated in vacuo. Silica gel chromatography (95:5 to 90:10 hexanes/EtOAc) afforded the title compound as a mixture of *cis* and *trans* acetonide diastereomers, small portions of which were separated by chromatography for characterization (7.55 g, 71%).

Cis acetonide (**67a**):

^{1}H NMR (500 MHz, $CDCl_3$): δ 4.80 (dd, J = 2.9, 12.1 Hz, 1H); 3.96 (dddd, J = 2.6, 4.8, 6.9, 9.3 Hz, 1H); 3.79 (dd, J = 4.8, 9.9 Hz, 1H); 3.58 (dd, J = 6.7, 9.9 Hz, 1H); 2.02 (dt, J_d = 13.0 Hz, J_t = 2.7 Hz, 1H); 1.85 (m, 1H); 1.47 (s, 3H); 1.44 (s, 3H); 1.07 (m, 21H).

^{13}C NMR (125 MHz, $CDCl_3$): δ 118.2, 100.1, 69.0, 66.5, 59.3, 32.1, 29.6, 19.4, 18.1, 12.1.

IR (NaCl plate, thin film, cm^{-1}): 2996, 2944, 2892, 2867, 1464, 1383, 1259, 1205, 1165, 1144, 1123, 1057, 1007, 882, 772, 683.

HRMS-ESI (m/z): $[M + Na]^+$ calculated for $C_{17}H_{33}NO_3Si$, 350.2122; found, 350.2125.

$[\alpha]_D$ = –5.3 (c = 1.4, $CHCl_3$, 24 °C).

Trans acetonide (**67b**):

^{1}H NMR (500 MHz, $CDCl_3$): δ 4.89 (m, 1H); 4.23 (m, 1H); 3.82 (dd, J = 4.9, 10.1 Hz, 1H); 3.64 (dd, J = 6.0, 10.1 Hz, 1H); 1.98 (m, 2H); 1.70 (s, 3H); 1.39 (s, 3H); 1.07 (m, 21H).

^{13}C NMR (125 MHz, $CDCl_3$): δ 120.1, 100.9, 66.7, 66.3, 58.9, 30.6, 29.8, 22.1, 18.1, 12.1.

IR (NaCl plate, thin film, cm^{-1}): 2944, 2867, 1464, 1384, 1263, 1244, 1206, 1122, 989, 882, 787, 683.

HRMS-ESI (m/z): $[M + Na]^+$ calculated for, $C_{17}H_{33}NO_3Si$, 350.2122; found, 350.2135.

$[\alpha]_D$ = +16.7 (c = 0.48, $CHCl_3$, 24 °C).

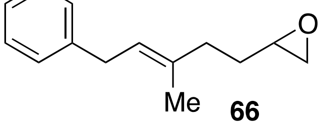

66

(*E*)-2-(3-Methyl-5-phenylpent-3-en-1-yl)oxirane (66): To NaH (60% dispersion in mineral oil, 0.457 g, 11.4 mmol, 1.1 equiv) was added pentane (10 mL). The mixture was stirred for 10 min at rt, then decanted. To the NaH was added DMSO (50 mL), then trimethylsulfoxonium iodide (2.63 g, 11.9 mmol, 1.15 equiv). The mixture was stirred 30 min at rt, then aldehyde **47** (1.95 g, 10.4 mmol, 1.0 equiv) was added. The reaction was stirred for an additional 30 min at rt, then for 3 h at 60 °C. The reaction was allowed to cool to rt, then was quenched with water (200 mL) and extracted 4× with Et_2O. The combined organics were washed once with water, then dried over $MgSO_4$, filtered, and concentrated in vacuo. Silica gel chromatography (95:5 to 90:10 hexanes/EtOAc) afforded the title compound as a pale yellow liquid (715.8 mg, 34%).

^{1}H NMR (500 MHz, $CDCl_3$): 7.29 (m, 2H); 7.19 (m, 3H); 5.41 (td, J_t = 7.3 Hz, J_d = 1.2 Hz, 1H); 3.38 (d, *J* = 7.3 Hz, 2H); 2.93 (m, 1H); 2.75 (dd, *J* = 4.3, 5.0 Hz, 1H); 2.48 (dd, *J* = 2.7, 5.0 Hz, 1H); 2.21 (m, 2H); 1.75 (s, 3H); 1.69 (td, J_t = 7.8 Hz, J_d = 5.7 Hz, 2H).

^{13}C NMR (125 MHz, $CDCl_3$): 141.7, 135.3, 128.6, 128.5, 126.0, 123.9, 52.3, 47.4, 36.0, 34.4, 31.2, 16.4.

IR (NaCl plate, thin film, cm^{-1}): 3027, 2979, 2920, 2853, 1603, 1494, 1453, 1410, 1384, 1261, 1074, 1030, 916, 837, 742, 699.

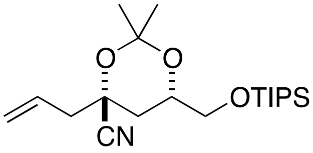

73

(4*S*,6*S*)-4-Allyl-2,2-dimethyl-6-(((triisopropylsilyl)oxy)methyl)-1,3-dioxane-4-carbonitrile (73): Diisopropylamine (2.67 mL, 19.1 mmol, 1.5 equiv) was dissolved in THF (20 mL) and cooled to –78 °C. To this was added *n*-butyllithium (2.5 M in hexanes, 7.63 mL, 19.1 mmol, 1.5 equiv) at –78 °C. The LDA solution was warmed to 0 °C in an ice bath for 10 minutes, then cooled again to –78 °C. In a separate reaction flask, nitrile **67** (4.17 g, 12.7 mmol, 1.0 equiv) was dissolved in THF (70 mL) and cooled to –78 °C. The LDA solution was added via cannula and the resultant reaction mixture stirred 30 min at –78 °C. Allyl bromide (2.20 mL, 25.4 mmol, 2.0 equiv) was filtered through a plug

of basic alumina and added to the reaction as a solution in THF (10 mL). The reaction was stirred for a further 2 h at –78 °C, then was quenched with sat. aq. NH_4Cl. After warming to rt, the aqueous layer was extracted three times with Et_2O. The combined organics were dried over $MgSO_4$, filtered, and concentrated in vacuo. Silica gel chromatography (95:5 to 90:10 hexanes/EtOAc) afforded the title compound as a pale yellow oil (2.46 g, 53%), along with recovered starting material (1.07 g).

^{1}H NMR (500 MHz, $CDCl_3$): δ 5.88 (dddd, *J* = 7.0, 7.5, 10.3, 17.4 Hz, 1H); 5.27 (m, 1H); 4.24 (m, 1H); 3.83 (dd, *J* = 4.9, 10.1 Hz, 1H); 3.63 (dd, *J* = 5.9, 10.1 Hz, 1H); 2.60 (dd, *J* = 7.0, 13.8 Hz, 1H); 2.51 (dd, *J* = 7.5, 13.8 Hz, 1H); 1.91 (dd, *J* = 2.1, 13.6 Hz, 1H); 1.72 (s, 3H); 1.58 (dd, *J* = 11.7, 13.5 Hz, 1H); 1.40 (s, 3H); 1.08 (m, 21H).

^{13}C NMR (125 MHz, $CDCl_3$): δ 130.1, 121.8, 121.1, 101.1, 69.6, 67.5, 66.4, 46.9, 35.9, 30.8, 21.6, 18.1, 12.1.

IR (NaCl plate, thin film, cm^{-1}): 3082, 2944, 2867, 1644, 1464, 1384, 1253, 1207, 1150, 1123, 996, 921, 882, 802, 777, 682.

HRMS-ESI (*m*/*z*): $[M + Na]^+$ calculated for $C_{20}H_{37}NO_3Si$, 390.2435; found, 390.2434.

$[\alpha]_D$ = +18.3 (*c* = 0.82, $CHCl_3$, 24 °C).

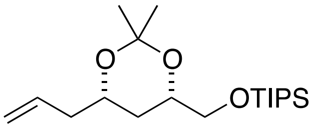

74

(((4*S*,6*S*)-6-Allyl-2,2-dimethyl-1,3-dioxan-4-yl)methoxy)triisopropylsilane (74): In a 50 mL round-bottom flask equipped with stir bar and dry ice condenser, nitrile **73** (2.30 g, 6.26 mmol, 1.0 equiv) was dissolved in a minimal amount of THF (5 mL) and cooled to –78 °C in a dry ice/acetone bath. Ammonia (~ 15 mL) was condensed into the flask. Freshly cut lithium wire (217 mg, 31.3 mmol, 5.0 equiv) was rinsed with hexanes and added to the reaction mixture. The dry ice bath was removed and the reaction heated under reflux for 30 min, as the substrate proved sparingly soluble at low temperatures. The reaction was quenched carefully with solid NH_4Cl then water until gas evolution ceased, then extracted three times with Et_2O. The combined organics

were dried over $MgSO_4$, filtered, and concentrated in vacuo. Silica gel chromatography (95:5 hexanes/EtOAc) afforded the title compound (1.40 g, 65%).

^{1}H NMR (500 MHz, $CDCl_3$): δ 5.83 (dddd, *J* = 6.7, 7.3, 10.2, 17.1 Hz, 1H); 5.08 (m, 2H); 3.93 (m, 2H); 3.77 (dd, *J* = 5.1, 9.7 Hz, 1H); 3.54 (dd, *J* = 6.7, 9.7 Hz, 1H); 2.33 (m, 1H); 2.19 (m, 1H); 1.71 (dt, J_t = 2.5 Hz, J_d = 12.9 Hz, 1H); 1.46 (s, 3H); 1.39 (s, 3H); 1.07 (m, 21H).

^{13}C NMR (125 MHz, $CDCl_3$): δ 134.5, 117.2, 98.7, 70.2, 68.8, 67.5, 41.2, 34.0, 30.3, 20.0, 18.2, 12.2.

IR (NaCl plate, thin film, cm^{-1}): 3078, 2993, 2943, 2867, 1643, 1464, 1380, 1262, 1201, 1173, 1116, 995, 916, 883, 800, 770, 682.

HRMS-ESI (*m*/*z*): $[M + H]^+$ calculated for $C_{19}H_{38}O_3Si$, 343.2663; found, 343.2674.

$[\alpha]_D$ = –16.2 (*c* = 0.99, $CHCl_3$, 23 °C).

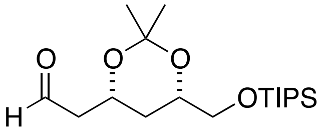

75

2-((4*R*,6*S*)-2,2-Dimethyl-6-(((triisopropylsilyl)oxy)methyl)-1,3-dioxan-4-yl)acetaldehyde (75): Alkene **74** (248 mg, 0.72 mmol, 1.0 equiv) was dissolved in CH_2Cl_2 (7.2 mL) and cooled to –78 °C. Ozone was bubbled through the solution for 1h, or until the solution appeared blue in color. Nitrogen was bubbled through the solution briefly to purge, then triphenylphosphine (285 mg, 1.09 mmol, 1.5 equiv) was added at −78 °C. Following the addition of PPh_3, the reaction mixture was allowed to warm to rt and was then concentrated in vacuo. Silica gel chromatography (95:5 to 90:10 hexanes/EtOAc) afforded the title compound as a colorless oil (227 mg, 91%).

^{1}H NMR (500 MHz, $CDCl_3$): δ 9.80 (dd, *J* = 1.7, 2.3 Hz, 1H); 4.45 (dddd, *J* = 2.5, 4.7, 7.4, 11.9 Hz, 1H); 4.00 (dddd, *J* = 2.5, 5.0, 6.9, 11.5 Hz, 1H); 3.78 (dd, *J* = 5.0, 9.8 Hz, 1H); 3.55 (dd, *J* = 6.7, 9.8 Hz, 1H); 2.64 (ddd, *J* = 2.4, 7.5, 16.6 Hz, 1H); 2.51 (ddd, *J* = 1.7, 4.7, 16.6 Hz, 1H); 1.76 (dt, J_t = 2.5 Hz, J_d = 12.8 Hz, 1H); 1.49 (s, 3H); 1.37 (s, 3H); 1.27 (ddd, *J* = 11.6, 11.6, 12.6 Hz, 1H); 1.07 (m, 21H).

^{13}C NMR (125 MHz, $CDCl_3$): δ 201.2, 98.9, 70.0, 67.1, 64.8, 50.1, 34.0, 30.1, 19.9, 18.1, 12.1.
IR (NaCl plate, thin film, cm^{-1}): 2993, 2944, 2893, 2867, 1728, 1464, 1382, 1262, 1201, 1170, 1126, 997, 882.
HRMS-ESI (*m*/*z*): $[M + Na]^+$ calculated for $C_{18}H_{36}O_4Si$, 367.2275; found, 367.2282.
$[\alpha]_D$ = –14.3 (*c* = 1.7, $CHCl_3$, 24 °C).

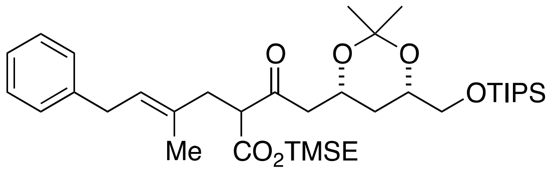

76

(*E*)-2-(Trimethylsilyl)ethyl 2-(2-((4*R*,6*S*)-2,2-dimethyl-6-(((triisopropylsilyl)oxy)methyl)-1,3-dioxan-4-yl)acetyl)-4-methyl-6-phenylhex-4-enoate (76): To a solution of diisopropylamine (378 μL, 2.69 mmol, 1.1 equiv) in THF (5 mL) at –78 °C was added *n*-butyllithium (2.5 M in hexanes, 1.08 mL, 2.69 mmol, 1.1 equiv). The mixture was warmed to 0 °C and stirred at that temperature for 10 min. The resulting LDA solution was added to a solution of ester **64** (820 mg, 2.69 mmol, 1.1 equiv) in THF (20 mL) at –78 °C. The reaction was stirred for 30 min at –78 °C, and a solution of aldehyde **75** (844 mg, 2.45 mmol, 1.0 equiv) in THF (5 mL) was added via cannula. The reaction was stirred for an additional 2 h at –78 °C. The reaction was quenched with saturated aq. NH_4Cl and extracted with Et_2O. The combined organics were dried over $MgSO_4$, filtered, and concentrated in vacuo. Silica gel chromatography (95:5 to 90:10 hexanes/EtOAc) afforded the aldol adduct (1.34 g, 84%) as a mixture of diastereomers, which was carried forward to the next step.

To a solution of aldol adduct (1.34 g, 2.07 mmol, 1.0 equiv) in CH_2Cl_2 were added 4 Å molecular sieves (500 mg), NMO (485 mg, 4.14 mmol, 2.0 equiv), and TPAP (73 mg, 0.21 mmol, 10 mol %). The reaction was stirred at rt for 2 h, then filtered through a plug of silica. The filtrate was concentrated in vacuo. Silica gel chromatography (90:10 hexanes/EtOAc) afforded the title compound as an undefined mixture of diastereomers at the central carbon of the β-ketoester (1.22 g, 91%).

^{1}H NMR (500 MHz, $CDCl_3$): δ 7.27 (m, 2H); 7.18 (m, 1H); 7.14 (m, 2H); 5.40 (tt, *J* = 1.4, 7.4 Hz, 1H); 4.37 (m, 1H); 4.14 (m, 2H); 3.95 (dddd, *J* = 2.6, 5.3, 6.4, 8.9 Hz, 1H); 3.73 (dd, *J* = 5.1, 9.9 Hz, 1H); 3.71 (m, 1H); 3.53 (dd, *J* = 6.4, 9.9 Hz, 1H); 3.33 (d, *J* = 7.3 Hz, 2H); 2.79 (app dt, 1H); 2.59 (m, 2H); 2.53 (m, 1H); 1.73 (app d, 3H); 1.70 (m, 1H); 1.44 (app d, 3H); 1.33 (s, 3H); 1.11 (m, 1H); 1.06 (m, 21H); 0.94 (m, 2H); 0.03 (app d, 9H).

^{13}C NMR (125 MHz, $CDCl_3$): δ 203.0, 167.7, 169.5, 141.2 (2 peaks), 132.5, 132.4, 128.6, 128.5, 126.0 (2 peaks), 125.9, 98.9, 70.0, 67.3, 65.7, 63.9, 58.9, 58.5, 48.7, 48.6, 37.8, 37.6, 34.4, 34.0, 33.9, 30.1, 19.9 (2 peaks), 18.1, 17.5 (2 peaks), 16.4, 16.3, 12.1 (2 peaks), –1.3 (2 peaks).

IR (NaCl plate, thin film, cm^{-1}): 2945, 2866, 1740, 1717, 1495, 1464, 1381, 1251, 1201, 1169, 1067, 993, 882, 860, 838, 697.

$[\alpha]_D$ = –12.2 (*c* = 0.57, $CHCl_3$, 24 °C).

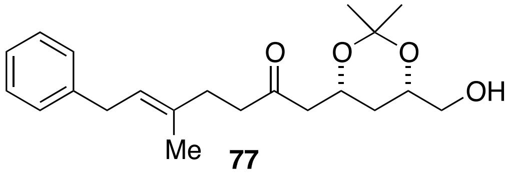

77

(*E*)-1-((4*R*,6*S*)-6-(Hydroxymethyl)-2,2-dimethyl-1,3-dioxan-4-yl)-5-methyl-7-phenylhept-5-en-2-one (77): To a solution of β-ketoester **76** (1.07 g, 1.66 mmol, 1.0 equiv) in THF (16 mL) was added TBAF (1 M in THF, 4.15 mL, 4.15 mmol, 2.5 equiv). The reaction was stirred at rt overnight, then quenched with saturated aq. NH_4Cl and extracted with Et_2O, then EtOAc. The combined organics were dried over $MgSO_4$, filtered, and concentrated in vacuo. Silica gel chromatography (1:1 hexanes/EtOAc) afforded the title compound as a colorless oil (409 mg, 71%).

^{1}H NMR (500 MHz, $CDCl_3$): δ 7.28 (m, 2H); 7.17 (m, 3H); 5.34 (td, J_t = 7.3 Hz, J_d = 1.2 Hz, 1H); 4.37 (m, 1H); 4.01 (dddd, *J* = 2.8, 2.8, 5.9, 5.9 Hz, 1H); 3.60 (m, 1H); 3.48 (m, 1H); 3.35 (d, *J* = 7.3 Hz, 2H); 2.69 (dd, *J* = 6.9, 16.2 Hz, 1H); 2.57 (app td, 2H); 2.43 (dd, *J* = 5.6, 16.2 Hz, 1H); 2.31 (t, *J* = 7.7 Hz, 2H); 1.98 (br, 1H); 1.72 (s, 3H); 1.48 (m, 1H); 1.46 (s, 3H); 1.37 (s, 3H); 1.27 (m, 1H).

^{13}C NMR (125 MHz, $CDCl_3$): δ 208.6, 141.6, 134.9, 128.6, 128.5, 126.0, 123.8, 99.1, 69.6, 66.1, 65.4, 49.3, 42.7, 34.4, 33.3, 32.2, 30.1, 20.0, 16.5.

IR (NaCl plate, thin film, cm^{-1}): 3471, 3027, 2993, 2915, 1713, 1494, 1453, 1381, 1268, 1201, 1168, 1127, 1059, 1028, 989, 911, 733, 699.

HRMS-ESI (*m*/*z*): $[M + H]^+$ calculated for $C_{21}H_{30}O_4$, 347.2217; found, 347.2230.

$[\alpha]_D$ = +1.5 (*c* = 1.68, $CHCl_3$, 24 °C).

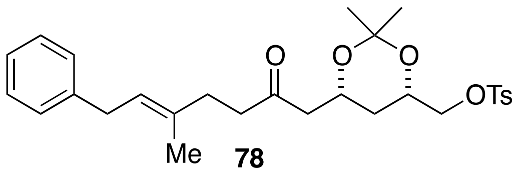

78

((4*S*,6*R*)-2,2-Dimethyl-6-((*E*)-5-methyl-2-oxo-7-phenylhept-5-en-1-yl)-1,3-dioxan-4-yl)methyl 4-methylbenzenesulfonate (78): To a solution of compound **77** (103.9 mg, 0.30 mmol, 1.0 equiv) in CH_2Cl_2 (2 mL) at 0 °C was added triethylamine (105 μL, 0.75 mmol, 2.5 equiv). In a small vial, tosyl chloride (91.5 mg, 0.48 mmol, 1.6 equiv) and Me_3NHCl (4.3 mg, 0.045 mmol, 15 mol %) were shaken in CH_2Cl_2 (1 mL) until homogeneous. This solution was then added to the reaction mixture dropwise via syringe over 5 min. The reaction was stirred for 1 h at 0 °C, then quenched with saturated aq. NH_4Cl. The organic phase was separated, and the aqueous phase washed with CH_2Cl_2. The organic extracts were washed with saturated aq. $NaHCO_3$, then brine. The combined organics were dried over $MgSO_4$, filtered, and concentrated in vacuo. Silica gel chromatography (80:20 to 2:1 hexanes/EtOAc) afforded the title compound (124.2 mg, 83%).

^1H NMR (500 MHz, $CDCl_3$): δ 7.79 (d, *J* = 8.3 Hz, 2H); 7.34 (d, *J* = 8.3 Hz, 2H); 7.28 (m, 2H); 7.17 (m, 3H); 5.33 (tq, J_t = 7.3 Hz, J_q = 1.3 Hz, 1H); 4.31 (m, 1H); 4.08 (m, 1H); 3.96 (dd, *J* = 5.8, 10.3 Hz, 1H); 3.90 (dd, *J* = 4.5, 10.3 Hz, 1H); 3.34 (d, *J* = 7.3 Hz, 2H); 2.65 (dd, *J* = 6.9, 16.3 Hz, 1H); 2.55 (app dt, 2H); 2.45 (s, 3H); 2.40 (dd, *J* = 5.5, 16.3 Hz, 1H); 2.30 (t, *J* = 7.6 Hz, 2H); 1.72 (s, 3H); 1.54 (dt, J_d = 12.7 Hz, J_t = 2.5 Hz, 1H); 1.37 (s, 3H); 1.27 (s, 3H); 1.13 (app q, 1H).

^{13}C NMR (125 MHz, $CDCl_3$): δ 208.3, 145.0, 141.5, 134.8, 133.1, 130.0, 128.6, 128.5, 128.2, 126.0, 123.8, 99.2, 72.3, 66.9, 65.1, 49.1, 42.6, 34.3, 33.3, 32.6, 29.9, 21.8, 19.7, 16.5.

IR (NaCl plate, thin film, cm^{-1}): 3061, 3027, 2992, 2917, 1715, 1599, 1495, 1453, 1363, 1264, 1177, 1098, 975, 816, 699, 667.

HRMS-ESI (*m*/*z*): $[M + Na]^+$ calculated for $C_{28}H_{36}O_6S$, 523.2125; found, 523.2131.

$[\alpha]_D$ = +2.0 (*c* = 2.33, $CHCl_3$, 24 °C).

References

1. Mohanty, S. S.; Uebelhart, P.; Eugster, C. H. *Helv. Chim. Acta* **2000**, *83*, 2036–2053. Dimethyl 1,3-acetonedicarboxylate was used as starting material.

2. Hayashi, Y.; Yamaguchi, H.; Toyoshima, M.; Okado, K.; Toyo, T.; Shoji, M. *Org. Lett.* **2008**, *10*, 1405–1408.

3. Arikan, F.; Li, J.; Menche, D. *Org. Lett.* **2008**, *10*, 3521–3524.

4. Rajesh, K.; Suresh, V.; Selvam, J. J. P.; Rao, C. B.; Venkateswarlu, Y. *Helv. Chem. Acta* **2009**, *92*, 1866–1872.

5. Ref. 4 reports $[\alpha]_D$ = 1.9 (*c* = 1, $CHCl_3$, 25 °C). The reason for the discrepancy is unclear; however, the literature and observed optical rotation values re-converge at the epoxy alcohol.

6. Zhao, J.-F.; Loh, T.-P. *Angew. Chem. Int. Ed.* **2009**, *48*, 7232–7235.

7. Guthrie, A. E.; Semple, J. E.; Joullié, M. M. *J. Org. Chem.* **1982**, *47*, 2369–2376.

8. Okino, T.; Hoashi, Y.; Takemoto, Y. *J. Am. Chem. Soc.* **2003**, *125*, 12672–12673.

9. Kaik, M.; Gawronski, J. *Tetrahedron: Asymmetry* **2003**, *14*, 1559–1563.

10. Berkessel, A.; Seelig, B. *Synthesis* **2009**, 2113–2115. A simplified synthesis avoiding preparation of this intermediate is presented.

11. Nicolaou, K. C.; Fylaktakidou, K. C.; Monenshein, H.; Li, Y.; Weyerhausen, B.; Mitchell, H. J.; Wei, H.-X.; Guntupalli, P.; Hepworth, D.; Sugita, K. *J. Am. Chem. Soc.* **2003**, *125*, 15433–15442.

12. Malkov, A. V.; Czemeryz, L.; Malyshev, D. A. *J. Org. Chem.* **2009**, *74*, 3350–3355.

13. Zhang, Y.; Phillips, A. J.; Sammakia, T. *Org. Lett.* **2004**, *6*, 23–25.

14. Crimmins, M. T.; Choy, A. L. *J. Am. Chem. Soc.* **1999**, *121*, 5653–5660.

15. Gaunt, M. J.; Jessiman, A. S.; Orsini, P.; Tanner, H. R.; Hook, D. F.; Ley, S. V. *Org. Lett.* **2003**, *5*, 4819–4822.

16. Saito, T. H. S.; Inaba, M.; Nishida, R.; Fujii, T.; Nomizu, S.; Moriwake, T. *Chem. Lett.* **1984**, *13*, 1389–1392.

17. Rychnovsky, S. D. *J. Org. Chem.* **1989**, *54*, 4982–4984.

18. Rychnovsky, S. D.; Zeller, S.; Skalitzky, D. J.; Griesgraber, G. *J. Org. Chem*. **1990**, *55*, 5550–5551.
